# Fit-for-purpose based testing and validation of antibodies to amino- and carboxy-terminal domains of cannabinoid receptor 1

**DOI:** 10.1007/s00418-021-02025-5

**Published:** 2021-08-27

**Authors:** Leyre Echeazarra, Gontzal García del Caño, Sergio Barrondo, Imanol González-Burguera, Miquel Saumell-Esnaola, Xabier Aretxabala, Maider López de Jesús, Leire Borrega-Román, Susana Mato, Catherine Ledent, Carlos Matute, María Aranzazu Goicolea, Joan Sallés

**Affiliations:** 1 Departament of Physiology, Faculty of Pharmacy, University of the Basque Country UPV/EHU, Vitoria-Gasteiz, Spain; 2 Department of Neurosciences, Faculty of Pharmacy, University of the Basque Country UPV/EHU, Vitoria-Gasteiz, Spain; 3 Department of Pharmacology, Faculty of Pharmacy, University of the Basque Country UPV/EHU, Vitoria-Gasteiz, Spain; 4 Department of Neurosciences, Faculty of Medicine and Nursing, University of the Basque Country UPV/EHU, Leioa, Spain; 5 Department of Analytical Chemistry, Faculty of Pharmacy, University of the Basque Country UPV/EHU, Vitoria-Gasteiz, Spain; 6 6IRIBHN, Universite Libre de Bruxelles, Brussels, Belgium; 7 Centro de Investigación Biomédica en Red de Salud Mental (CIBERSAM), 28029 Madrid, Spain; 8 Bioaraba, Neurofarmacología Celular y Molecular, 01008 Vitoria-Gasteiz, Spain; 9 Bioaraba, Dispositivos Móviles para el Control de Enfermedades Crónicas, 01008 Vitoria-Gasteiz, Spain; 10 Centro de Investigación Biomédica en Red sobre Enfermedades Neurodegenerativas (CIBERNED), Madrid, Spain; 11 Achucarro Basque Center for Neuroscience, Leioa, Spain; 12 Multiple Sclerosis and Other Demyelinating Diseases Unit, Biocruces Bizkaia, Barakaldo, Spain

**Keywords:** Antibody specificity, CB_1_ receptor, Carboxy-terminus, Amino-terminus, Antigen retrieval, CB_1_-knockout mice

## Abstract

Specific and selective anti-CB_1_ antibodies are among the most powerful research tools to unravel the complex biological processes mediated by the CB_1_ receptor in both physiological and pathological conditions. However, low performance of antibodies remains a major source of inconsistency between results from different laboratories. Using a variety of techniques, including some of the most commonly accepted ones for antibody specificity testing, we identified three of five commercial antibodies against different regions of CB_1_ receptor as the best choice for specific end-use purposes. Specifically, an antibody against a long fragment of the extracellular amino tail of CB_1_ receptor (but not one against a short sequence of the extreme amino-terminus) detected strong surface staining when applied to live cells, whereas two different antibodies against an identical fragment of the extreme carboxy-terminus of CB_1_ receptor (but not one against an upstream peptide) showed acceptable performance on all platforms, although they behaved differently in immunohistochemical assays depending on the tissue fixation procedure used and showed different specificity in Western blot assays, which made each of them particularly suitable for one of those techniques. Our results provide a framework to interpret past and future results derived from the use of different anti-CB_1_ antibodies in the context of current knowledge about the CB_1_ receptor at the molecular level, and highlight the need for an adequate validation for specific purposes, not only before antibodies are placed on the market, but also before the decision to discontinue them is made.

**Supplementary Information:**

The online version contains supplementary material available at 10.1007/s00418-021-02025-5.

## Introduction

The endogenous cannabinoid system is composed of endogenous ligands (endocannabinoids), such as anandamide (AEA) and 2-arachidonoylglycerol (2-AG), the enzymes responsible for their turnover and the inhibitory G-protein-coupled receptors (GPCRs) CB_1_ and CB_2_ (Piomelli 2003; Kano et al. 2009). CB_1_ receptor is the most abundant GPCR in the central nervous system (Herkenham 1991; Piomelli 2003) and is densely expressed in brain (Herkenham 1991; Mailleux and Vanderhaeghen 1992; Matsuda et al. 1993; Dove Pettit et al. 1998; Tsou et al. 1998; Marsicano and Lutz 1999; Egertová and Elphick 2000; Howlett et al. 2002; McPartland et al. 2007). It is now known that brain CB_1_ receptor plays key roles in regulating a variety of behavioural responses and primary physiological processes, such as memory and cognitive processes, motor activity, pain perception, temperature regulation, feeding behaviour, energy balance and stress responses (Maldonado et al. 2020), while dysregulation of CB_1_ receptor-mediated signalling underlies a plethora of pathological conditions, including neuropsychiatric and neurodegenerative diseases among others (Cristino et al. 2020). Thus, CB_1_ receptor has emerged as a promising therapeutic target for a variety of diseases (Chicca et al. 2017; Di Marzo 2018; Cristino et al. 2020; Fernández-Ruiz et al. 2020), and consequently, research towards the development of synthetic CB_1_ and natural ligands as potential therapeutic drugs for brain disorders underwent a rapid expansion (An et al. 2020; Cinar et al. 2020), in parallel with a growing effort of basic scientists towards unravelling the complex molecular mechanisms of CB_1_ receptor-mediated signalling. The expression of brain CB_1_ receptors in a variety of cell phenotypes and subcellular compartments, the pleiotropic effects of exogenous CB_1_ receptor ligands and the dynamic processes governing CB_1_ receptor trafficking (Busquets-Garcia et al. 2018) constitute additional sources of complexity that require the use of reliable research tools, of which specific and selective anti-CB_1_ antibodies are among the most powerful ones.

An important caveat for the use of antibodies is that they may provide poorly reproducible and inaccurate results, and therefore, antibody testing and validation are essential before being used in research. Development of reliable antibodies against GPCRs is especially challenging (Saper 2005; Jositsch et al. 2009; Kirkpatrick 2009; Talmont et al. 2012; Baker 2015), and serious doubts had been raised about the usefulness of a variety of anti-GPCR antibodies (O’Connell et al. 2006; Rhodes and Trimmer 2006; Pradidarcheep et al. 2008; Jositsch et al. 2009; Michel et al. 2009). Obviously, all these caveats are equally applicable to antibodies against CB_1_ receptor, and proper validation is a fundamental pre-requisite before studies using these antibodies are conducted. However, there are only two research papers devoted entirely to the study of the specificity of anti-CB_1_ antibodies. In one of these studies (Grimsey et al. 2008), five antibodies generated against different sequences of the amino- and carboxy-tails of the CB_1_ receptor were tested for specificity by immunohistochemistry, in tissue sections of mouse brain and transfected HEK cells, and by Western blot, in transfected cells and brain lysates. The authors reported good results for two antibodies developed by Ken Mackie’s research group (Hájos et al. 2000; Wager-Miller et al. 2002) against carboxy-terminal (C-terminal) cytosolic regions of the CB_1_ receptor, but poor specificity for three commercial antibodies against amino-terminal (N-terminal) extracellular regions of CB_1_ receptor in all end uses assayed. In a more recent study using two commercial N-terminal and two C-terminal antibodies, authors focused on establishing the appropriate conditions for Western blot detection and immunoprecipitation of CB_1_ receptor in samples from brain and cortical neuron cultures (Esteban et al. 2020). This study emphasized the importance of temperature and detergents for the final result and proposed a new interpretation of Western blot and immunoprecipitation data based on the folding and packing state of CB_1_ and the detergent used.

Notably, antibody testing and validation must consider their end-use application, and a recently proposed guide for antibody validation included explicit recommendations on the suitability experimental approaches for such a purpose (Uhlen et al. 2016). Circumventing this aspect can lead companies to discontinue production of antibodies that would otherwise be very useful for a given platform, and indeed, several companies have incorporated the fit-for-purpose (F4P) concept for antibody development (Voskuil 2014). Here we performed a F4P-based analysis of the specificity of five representative commercial anti-CB_1_ antibodies designed against N- and C-terminal regions of CB1 receptor (hereinafter referred to as N- and C-terminal antibodies) and selected on the basis of the sequences against which they were generated, which can determine the final outcome in different end-use applications. This included two N-terminal and one C-terminal antibodies from Santa Cruz Biotechnology, which have been discontinued and replaced by other antibodies probably due to low demand, and two polyclonal antibodies raised in goat and rabbit against the 31 amino acids at the extreme carboxy-terminus (C-terminus) of CB_1_ receptor, which have been widely used in the last decade (Yoneda et al. 2013; Rivera et al. 2015; Rodríguez-Cueto et al. 2016; Mateo et al. 2017; Puighermanal et al. 2017; Rhomberg et al. 2018; Diniz et al. 2019; Puente et al. 2019; Uchigashima et al. 2020; Exposito-Alonso et al. 2020; Peñasco et al. 2020; Egaña-Huguet et al. 2021; Fuerte-Hortigón et al. 2021) and validated for some applications using different transgenic mice models lacking CB_1_ receptor, either completely or in specific cell phenotypes or subcellular compartments (Hebert-Chatelain et al. 2014a; Remmers et al. 2017; Gutiérrez-Rodríguez et al. 2018). To this end, our workflow combined commonly accepted testing and validation approaches along with pharmacological assays to confirm or rule out the presence of CB_1_ receptor in samples yielding CB_1_-like immunoreactive bands on Western blot. Of the five antibodies analysed, only the two raised against the extreme C-terminus of CB_1_ were suitable for detection of CB_1_ receptor in all the applications tested. However, although the other three antibodies analysed were unable to detect CB_1_ receptor by Western blot and by immunohistochemistry in tissue sections, two of them recognized CB_1_ receptor in CB_1_-transfected cells HEK-293 cells, and moreover, one of them raised against a large fragment of the extracellular N-terminal region of CB_1_ receptor yielded strong specific immunofluorescence at the plasma membrane under non-permeabilizing conditions in live cells. Our results provide robust data on the suitability for different applications of the anti-CB_1_ antibodies tested, and highlight the importance of choosing the platform that best fits the end use of a given antibody before discarding it for any use on the basis of an inaccurate validation approach.

## Materials and methods

### Animals

Sprague–Dawley rats were obtained from SGIker facilities (University of the Basque Country, UPV/EHU, Spain). CB_1_ receptor null mutant (CB_1_-KO) and wild-type (CB_1_-WT) mice were either bred from the Spanish colony established at the University of the Basque Country (Ledent’s CB_1_-KO mice) or kindly provided by Dr. Giovanni Marsicano (Institute François Magendie, Bordeaux, France) (Marsicano’s CB_1_-KO mice) and genotyped as described before (Ledent et al. 1999; Marsicano et al. 2002). Both mice and rats were kept in a controlled environment (12 h light–dark cycle, 22 ± 2 °C and 55 ± 5% relative humidity) with food and water provided ad libitum, for at least 7 days until they were sacrificed at 10–12 weeks of age. All experiments involving animals were approved by the Committee of Ethics for Animal Welfare of the University of the Basque Country (UPV/EHU; CEBA/146/2010 and CEBA/61/2010) and performed following guidelines of the Directive of the European Commission (2010/63/EU) and Spanish regulations (RD 53/2013) for care and management of experimental animals.

### Perfusion and preparation of tissue sections for immunohistochemistry

Six adult Sprague–Dawley rats, four adult mice from the Ledent’s line (2 CB_1_-WT and 2 CB_1_-KO) and four adult mice from the Marsicano’s line (2 CB_1_-WT and 2 CB_1_-KO) were used for immunohistochemistry. Animals were anaesthetized intraperitoneally with an overdose of choral hydrate (1 g/kg i.p.; Panreac Química S.A., Castellar del Vallés, Barcelona, Spain) before perfusion. Rats were transcardially perfused with either 0.1 M phosphate-buffered saline pH 7.4 (PBS) or a 0.37% (w/v) sulphide solution (three animals each) for 4 min, followed by 4% (w/v) paraformaldehyde (Sigma, St. Louis, MO, USA) in 0.1 M phosphate buffer for 4 min at a constant flow of 30 ml/min (Heidolph Instruments GmbH & Co. KG, Pumpdrive PD 5106, Schwabach, Germany). All four CB_1_-WT and CB_1_-KO mice were transcardially perfused with a 0.37% (w/v) sulphide solution for 4 min, followed by 4% (w/v) paraformaldehyde for 4 min at a constant flow of 10 ml/min. After that, brains were removed and kept immersed in the same fixative medium during 4 h. Next, brains were transferred to phosphate buffer 0.1 M, pH 7.4 (PB) containing 30% sucrose and kept at 4 °C and constant stirring until they sank. Brains were cryosectioned using a microtome (Leitz-Wetzlar 1310, Wetzlar, Germany) provided with a specific sensor to control temperature (5MP BFS-Physitemp Controller, Clifton, New Jersey, USA). Twelve (rats) or six (mice) separate representative series of free-floating 40-µm-thick coronal sections were obtained from each brain and collected in PBS. Sections were cryoprotected by incubations in increasing concentrations (5%, 10% and 20% v/v) of dimethyl sulphoxide (Sigma, St. Louis, MO, USA) in PB. Section series were then separately placed in the bottom of Eppendorf tubes, subjected to a permeabilization protocol, consisting of three freeze–thaw cycles in isopentane at −80 °C, and stored frozen until use.

### Cell culture and transfection of HEK-293 cells

Human embryonic kidney 293 (HEK-293) from the American Type Culture Collection (ATCC; CRL-1573^™^) were grown in 75 cm^2^ cell culture flasks (430,725; Corning, Barcelona, Spain) in DMEM culture medium (ATCC, 30–2002), supplemented with 10% fetal bovine serum (Sigma-Aldrich) and antibiotics (100 U/mL penicillin and 100 µg/mL streptomycin, Gibco, Life Technologies S.A., Madrid, Spain). When approaching 70–80% confluence, cells were harvested using trypsin–EDTA solution (25,300–054, Gibco, Barcelona, Spain) and transferred to 12-well plates containing poly-d-lysine coated glass coverslips. When they reached 70–80% confluence, cells were transfected with pcDNA3.0 plasmid containing a cDNA insert encoding the human cannabinoid receptor 1 (pCDNA-CB_1_; 1 µg DNA/well) using Lipofectamine 3000 (L3000001; Invitrogen S.A., Spain). Cells were processed for single or double immunofluorescence 48 h after transfection.

### Isolation of enriched subcellular fractions

A total of ten adult Sprague–Dawley rats and ten adult mice from the Ledent’s line (5 CB_1_-WT and 5 CB_1_-KO) were used for isolation of subcellular fractions intended to be used in Western blot and pharmacological assays. After sacrificing animals by decapitation, brains were immediately removed and cerebral cortices were dissected out on ice and stored at −80 °C. P1, P2 and cytosolic (Cyt) subcellular fractions from five rat and four mouse (5 CB_1_-WT and 5 CB_1_-KO) cerebral cortex samples were obtained essentially as previously described for rat and human brain tissues (Garro et al. 2001; Sallés et al. 2001; Montaña et al. 2012; García del Caño et al. 2015). To isolate highly purified intact nuclei (N fraction) used for both immunofluorescence and Western blot, we followed the procedure described by Thompson and colleagues (Thompson 1973) with slight modifications (Montaña et al. 2012; García del Caño et al. 2015) (Supplementary Material and Methods for details).

### Immunohistochemistry and double immunofluorescence

Brain sections were incubated free-floating with the same amount of freshly prepared reaction solutions in all cases. Sections were treated for 20 min with 1% H_2_O_2_ in phosphate-buffered saline 0.1 M, pH 7.4 (PBS) to inactivate endogenous peroxidase. Thereafter, they were incubated at 20–25 °C for 1 h in blocking solution, consisting of PBS containing 1% serum albumin bovine (BSA; Sigma, St. Louis, MO, USA) and 1% normal goat or rabbit serum (Vector Laboratories, Burlingame, CA, USA) for anti-CB_1_ antibodies raised in rabbit (H150 and Af380) or goat (N15, K15 and Af450), respectively (see details for anti-CB_1_ receptor antibodies in Table 1). Subsequently, tissue sections were incubated overnight at 4 °C in the corresponding anti-CB_1_ primary antibody diluted in blocking solution. Sections were then incubated for 1 h at 20–25 °C with affinity-purified biotinylated secondary antibodies goat anti-rabbit (BA-1000; Vector Laboratories) or rabbit anti-goat (BA-5000; Vector Laboratories), both diluted 1:200 in blocking solution. Sections were then processed by the avidin–biotin-peroxidase method using the Vectastain kit (Vector Laboratories) and reacted with 0.05% 3,3′-diaminobenzidine tetrahydrochloride and 0.01% H_2_O_2_ in 50 mM Tris-HCl, pH 7.6. Finally, the sections were mounted onto gelatine-coated slides, air-dried, dehydrated and coverslipped using DPX (Fluka, Buchs, Switzerland).

**Table 1:** Anti-CB_1_ receptor primary antibodies

Short name	Dilution (IHC/IF)	Dilution (WB)	Host and clonality	Isotype and purity	Immunizing antigen	Source, cat. no.
N15	1:100	1:500	Goat polyclonal	Affinity-purified IgG	Peptide derived from within residues 1–50 of the human CB_1_ receptor	Santa Cruz Biotech., CB1 (N-15): sc-10066
H150	1:200	1:250	Rabbit polyclonal	Affinity-purified IgG	Peptide corresponding to amino acids 1–150 of human CB_1_ receptor	Santa Cruz Biotech., CB1 (H-150): sc-20754
K15	1:200	1:250	Goat polyclonal	Affinity-purified IgG	Peptide sequence from within residues 397–447 of the human CB_1_ receptor	Santa Cruz Biotech., CB1 (K-15): sc-10068
Af380	1:200	1:1000	Rabbit polyclonal	Immunogen affinity-purified IgG	Peptide corresponding to the carboxy-terminal 31 amino acids of mouse CB1 receptor	Frontier Institute Co., Ltd., CB1-Rb-Af380
Af450	1:200	1:500	Goat polyclonal	Immunogen affinity-purified IgG	Peptide corresponding to the carboxy-terminal 31 amino acids of mouse CB_1_ receptor	Frontier Institute Co., Ltd., CB1-Go-Af450

Antibody manufacturers: Santa Cruz Biotechnology, Santa Cruz, CA, USA; Frontier Science Co. Ltd., Hokkaido, Japan

*IHC* immunohistochemistry, *IF* immunofluorescence, *WB* Western blot

For double immunofluorescence in tissue, sections were preincubated at 20–25 °C for 1 h in blocking solution, consisting of 1% serum albumin bovine (BSA; Sigma, St. Louis, MO, USA) and 1% normal donkey serum (Jackson Immunoresearch Laboratories, Inc.; West Grove, PA, USA), followed by overnight incubation at 4 °C with a combination of goat anti-CB_1_ Af450 and mouse monoclonal anti-LaminB1 (sc-56144; Santa Cruz Biotechnology) primary antibodies, diluted in blocking solution, both at a final concentration of 2 µg/ml. Thereafter, sections were incubated at 20–25 °C temperature for 1 h in Alexa Fluor 488 donkey anti-goat IgG (A11055; Invitrogen S.A.) and DyLight 549 donkey anti-mouse F(ab′)2 fragment (715-506-151; Jackson Immunoresearch Laboratories, Inc.) both diluted 1:400 in blocking solution. Finally, sections were mounted onto gelatine-coated slides with Mowiol reagent (Calbiochem, Bad Soden am Taunus, Germany). When the immunizing peptide was available (N15, K15, Af380 and Af450), negative controls were performed by using antibodies preabsorbed overnight at 4 °C with excess immunizing antigen (IgG-to-peptide mass ratios 1:5 in all cases).

Cells processed for single or double immunofluorescence against CB_1_ receptor under permeabilizing conditions were fixed for 10 min at 20–25 °C with 4% phosphate-buffered paraformaldehyde, washed extensively with wash buffer (PBS containing 0.22% gelatine) and incubated with permeabilizing blocking buffer (PERM; wash buffer containing 0.066% saponin, 1% bovine serum albumin, 1% normal donkey serum) for 1 h at 20–25 °C. Thereafter, cells were incubated overnight at 4 °C with rabbit (H150 and Af380) or goat (N15, K15 and Af450) anti-CB_1_ primary antisera (see Table 1 for details), either alone (for single immunofluorescence) or in the following combinations (for double immunofluorescence): Af380/N15 Af450/H150, Af380/K15 or Af450/Af380. To test the ability of N-terminal antibodies to detect surface CB_1_ receptor in live HEK-293 cells, culture medium was replaced with cold serum-free OptiMEM (51,985–034; Life Technologies, Barcelona, Spain), and goat N15 or rabbit H150 antibodies were added directly to cell cultures, followed by incubation for 30 min at 4 °C. After three washes with cold PBS, cells were fixed, washed and blocked as above and incubated overnight at 4 °C with Af380 or Af450 antibodies, respectively. After three washes (10 min each) at 20–25 °C with washing buffer, cells were incubated with the appropriate fluorescent dye-conjugated secondary antibodies diluted 1:400 in PERM, for 1 h at 20–25 °C. Thus, Dylight 549 Donkey anti-Rabbit F(ab′)2 fragment (711-506-152, Jackson Immunoresearch Laboratories, Inc.; West Grove, PA, USA,) and Alexa Fluor 488 Donkey anti-Goat IgG (A-11055; Invitrogen S.A.) were used either alone, for single immunofluorescence, or combined, for double immunofluorescence. After the secondary antibody incubation, cells were washed twice with wash buffer for 10 min at 20–25 °C, and cell nuclei were counterstained with 0.1 µg/mL Hoechst 33,342 (Sigma-Aldrich) in wash buffer, for 10 min at 20–25 °C. After two additional washes (10 min each) at 20–25 °C with PBS, cells were mounted onto glass slides using homemade Mowiol (Calbiochem, Madrid, Spain) mounting medium, containing anti-fade reagent 1,4-phenylene-diamine dihydrochloride (Sigma-Aldrich).

Double immunofluorescence in intact nuclei with anti-CB_1_ Af450 antibody combined with mouse monoclonal antibodies to the nuclear components Lamin-B1, Histone H1, NeuN-Fox-3 or SC35 (see Table S1 for details) was performed as described for fixed HEK-293 cells, except that Alexa Fluor 488 donkey anti-goat IgG (A-11055; Invitrogen S.A.) and DyLight 549 donkey anti-mouse F(ab′)2 fragment (715-506-151; Jackson Immunoresearch Laboratories, Inc.) were used as secondary antibodies.

To establish dilutions of the primary antibodies used in the immunohistochemistry and double immunofluorescence experiments (Table 1), preliminary tests were carried out at three dilutions in transfected HEK-293 cells and the one that provided the best signal-to-noise ratio was chosen. The concentrations tested for all antibodies were 1: 100, 1: 200 and 1: 400, which were based on the range recommended by the manufacturer in the case of Santa Cruz antibodies N15, H150 and K15 (1: 50–1: 500) and on the data available in the literature in the case of Frontier antibodies. Institute Co. Af380 and Af450.

### Microscope imaging and co-localization analysis

Immunostained brain sections were examined with an Olympus BX50F optic microscope (Olympus, Tokyo, Japan) equipped with a high-resolution digital camera (Olympus and Soft Imaging Systems, Tokyo, Japan). Images were digitized using CellA software for image acquisition with automatic or manual exposure control (Olympus and Soft Imaging Systems, Tokyo, Japan). Images of double immunofluorescence-stained tissue sections were captured sequentially on an Olympus Fluoview FV500 confocal microscope (Olympus, Tokyo, Japan) equipped with a diode laser line of 405 nm, an Argon laser line of 457, 488 and 514 nm, and HeNe laser line of 543 nm and 633 nm. Alexa Fluor 488 was viewed using 505/525 nm BP filters and Alexa Fluor 568 using 560–600 nm BP filters. Images were acquired using a pinhole of one airy unit and objective 60× (1.40 NA, Plan Apochromat). Viewing of *Z*-stacks and minor despeckling was performed on the Fluoview Image Browser software, version 5.0 (Olympus, Tokyo, Japan). Images were subsequently exported to TIFF format. Boundaries of cortical layers were determined on the basis of variations in the intensity of the immunohistochemical reaction and Nissl staining distribution in neighbouring sections. All figures were compiled and labelled using Adobe Photoshop CS3.

Fluorescence imaging of CB_1_-transfected HEK-293 cells was performed on a Carl Zeiss Axio Observer.Z1 epifluorescence microscope (Carl Zeiss MicroImaging, Inc, Gottigen, Germany), equipped with a HXP 120 C metal halide light source. Micrographs were acquired using a AxioCam MRm (1388 × 1040 pixels) monochromatic camera (Carl Zeiss MicroImaging, Inc.) and a 63× Plan-Apochromat objective (NA 1.4) with exposure time set to levels just below saturation for each dye. The ApoTome structured illumination module and a computer-controlled *XYZ* motorized stage (both from Carl Zeiss MicroImaging, Inc.) were used to obtain optical sections in the *Z*-axis, with camera settings adjusted to obtain images with a pixel size of 0.01 µm^2^. Bandpass filters used were 49 DAPI (Ex G 365/Em 445/50) for Hoechst’s staining, 38 HE eGFP (Ex 470/40, Em 525/50) for Alexa Fluor 488, and 43 HE Cy3 shift free (Ex 550/25, Em 605/70) for DyLight 549. Images were digitized using Zeiss Axio Vision 4.8 software (Carl Zeiss MicroImaging, Inc). Minor despeckling was performed on ImageJ (NIH, Bethesda, MD, USA) software. Images were exported to TIFF format, and compiled and labelled using Adobe Photoshop CS3 (San Jose, CA, USA).

Co-localization analysis was performed on 10–14 representative images for each of the four antibody combinations (Af380/N15, *n* = 10; Af450/H150, *n* = 11; Af380/K15, *n* = 14; Af380/Af450, *n* = 11) from at least two independent experiments. To calculate Mander’s overlap (M1 and M2) and Pearson’s intensity correlation (*R*_*P*_) coefficients, all *Z*-stacks acquired from the top to the bottom of cells at 0.24 µm intervals were analysed using JACoP plugin (Bolte and Cordelières, 2006) in Fiji-ImageJ Software (National Institute of Health, Bethesda, MA, USA). A Kruskal–Wallis analysis of variance was used to identify statistically significant differences in Mander’s and Pearson’s coefficients among the four antibody combinations. Dunn’s multiple comparison post hoc test was used to find significant differences between selected pairs of antibody combinations. The level of significance was set at *P* < 0.05 for both analyses.

### Western blotting

Western blot studies were performed as previously reported with minor modifications (Garro et al., 2001; López de Jesús et al., 2006; Ruiz de Azúa et al. 2006). Briefly, known amounts of total protein from P1, P2, Cyt or N fractions were heated for 5 min at 60 °C in urea-denaturing buffer (20 mM Tris-HCl, pH 8.0, 12% glycerol, 12% urea, 5% dithiothreitol, 2% sodium dodecyl sulphate, 0.01% bromophenol blue) and resolved by electrophoresis in 5–12% gradient SDS-polyacrylamide gels (SDS-PAGE) using the Mini Protean II gel apparatus (Bio-Rad; Hercules, CA, USA). Proteins were transferred to polyvinylidene fluoride (PVDF) membranes (Amersham Biosciences, Piscataway, NJ, USA), using the Mini TransBlot transfer unit (Bio-Rad; Hercules, CA, USA) at 30 V constant overnight at 4 °C and processed for immunoblot analysis (see Supplementary Material and Methods for details).

### Binding assays

Radioligand binding assays were carried out as previously described with minor modifications (López-Rodríguez et al. 2002; Barrondo and Sallés 2009; Casadó et al. 2010). The affinity (*K*_*D*_) and the maximal number of sites (*B*_*max*_) for the selective CB_1_ receptor antagonist [^3^H]SR141716A were measured by saturation binding experiments in P1, P2 and N subcellular fractions of the adult rat brain cortex (see Supplementary Material and Methods for details). For assays of [^35^S]GTPγS binding stimulated by increasing concentrations of the CB_1_ receptor agonist WIN 55,212-2, we followed the procedure described elsewhere for human and rat brain membranes (González-Maeso et al. 2000; Barrondo and Sallés, 2009) to obtain the efficacy (*E*_*max*_) and potency (*EC*_*50*_) values.

Radioligand and [^35^S]GTPγS binding assays were performed in triplicate and duplicate, respectively, and the results were obtained from at least three independent experiments. Data are expressed as mean ± SEM. Experimental data were analysed using computerized iterative procedure (GraphPad Prism version 4.0) by directly fitting the data to the suitable mathematical models, as described previously (Barrondo and Sallés 2009; Casadó et al. 2010). For the statistical significance (set at *P* < 0.05) of the differences between affinity and potency constant values, these parameters were logarithmically transformed because it has been demonstrated that parameters like affinity and EC_50_ constants obtained experimentally are log-normally distributed, and, therefore, statistical analysis should be performed as such (Christopoulos 1998) (see Supplementary Material and Methods for further details).

## Results

**Fig. 1:**
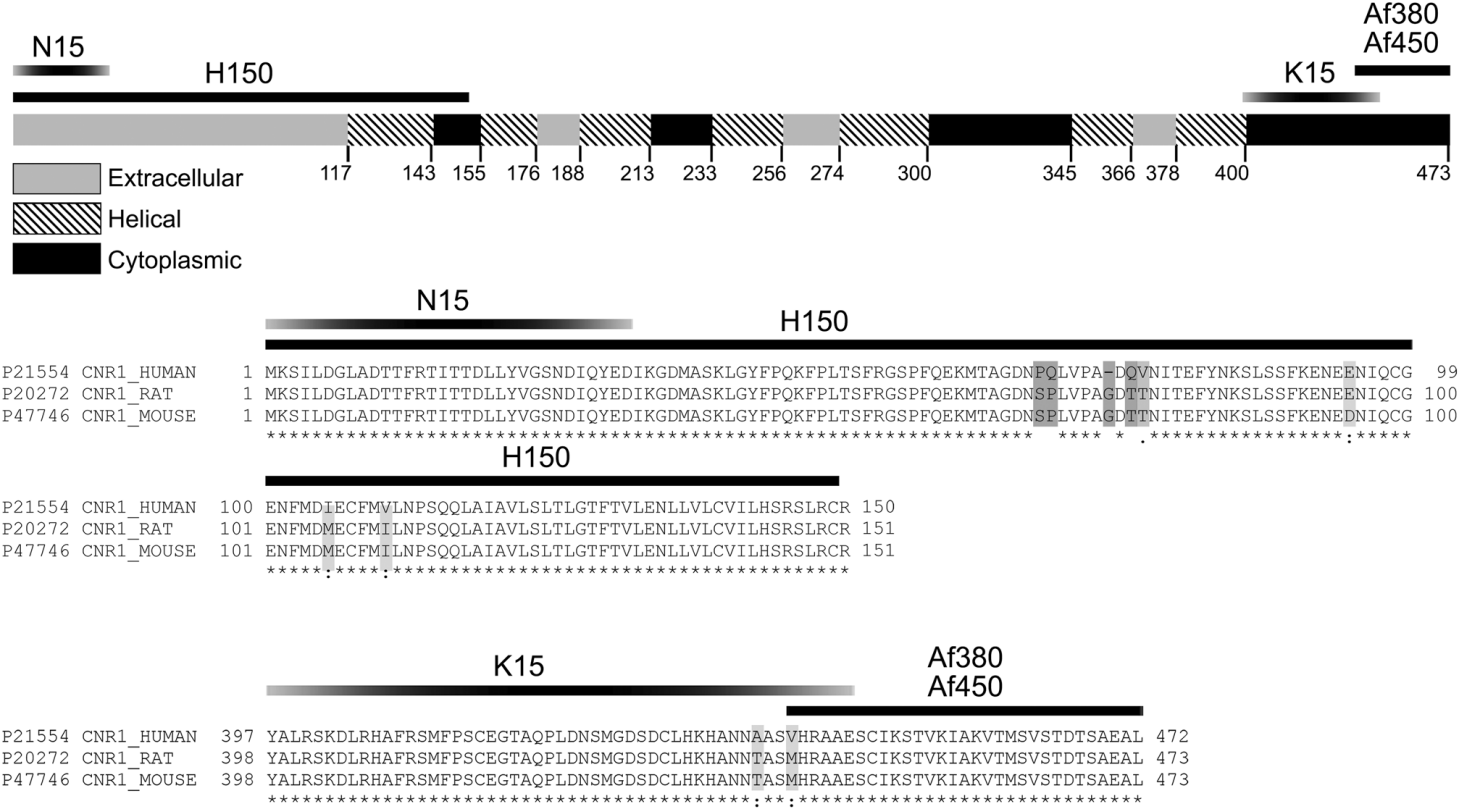
Linear scale representation of the sequence of the rat CB_1_ receptor (Uniprot ID P20272; available at http://www.uniprot.org/) and amino acid sequence alignment of human, rat and mouse CB_1_ receptor using UniProt Align (https://www.uniprot.org/align/). The position of the antigenic sequences used to produce the anti-CB_1_ rabbit (H150, Af380) and goat (N15, K15, Af450) polyclonal antibodies tested are indicated in both (see Table 1 for further details on anti-CB_1_ antibodies). Numbers on the linear representation of CB_1_ receptor refer to the amino acid residues in the sequence. Asterisks, two dots and one dot below the sequence alignment indicate fully conserved, highly conserved and weakly conserved residues across human, rat and mouse.

Five commercial antibodies designed against different sequences of the CB_1_ receptor highly conserved across human mouse and rat were assayed for specificity. Goat polyclonal antibodies N15 and H150 were designed against two different sequences of the N-terminal region of the human CB_1_ receptor. Rabbit polyclonal antibody K15 was raised against an internal sequence near the C-terminus of CB_1_ receptor. Af380 and Af450 antibodies were both raised against the C-terminal 31 amino acids of CB_1_ receptor, and produced in rabbit and goat, respectively (Fig. 1; Table 1).

**Fig. 2:**
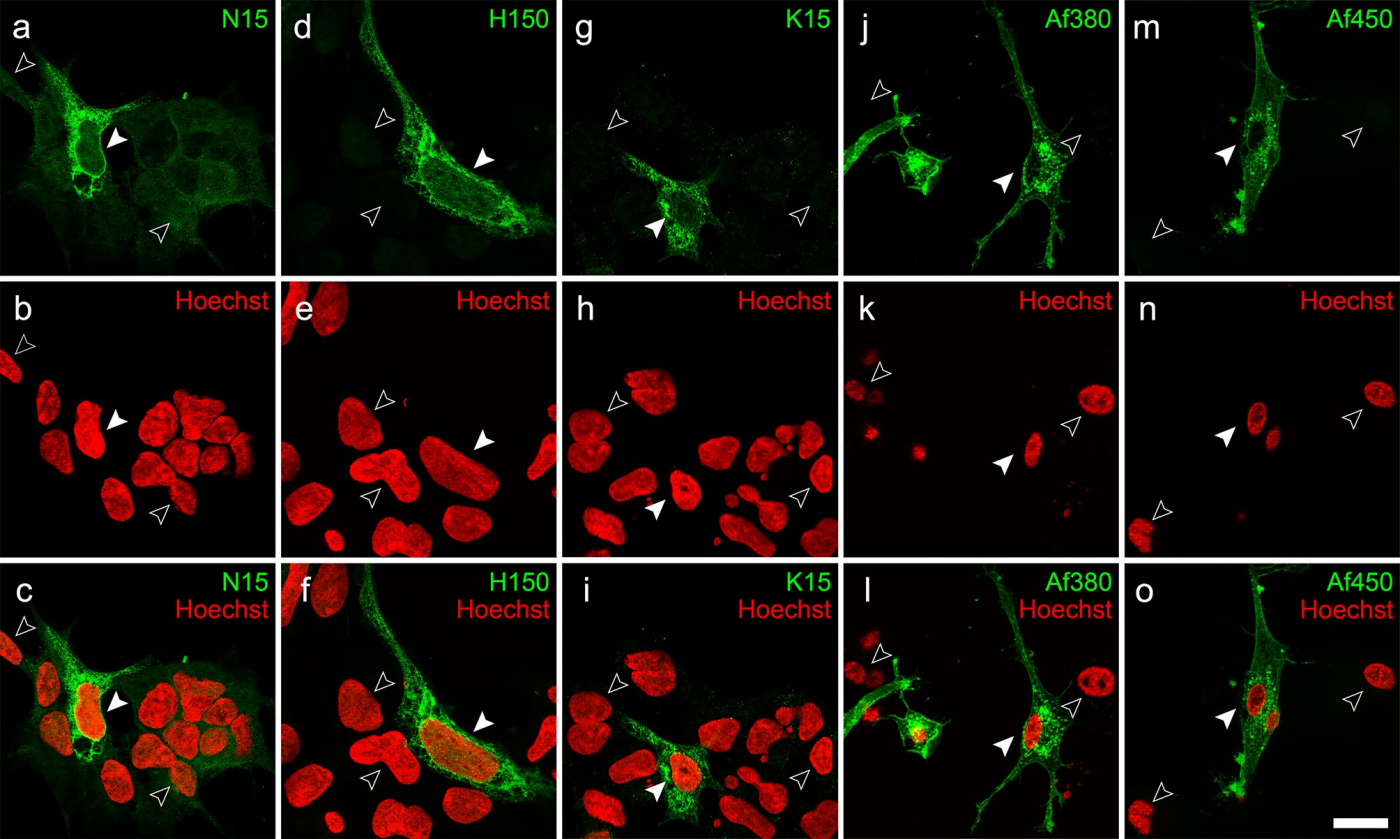
Immunofluorescence labelling of CB_1_-transfected HEK293 cells (pseudocoloured green) with anti-CB_1_ polyclonal antibodies N15 (**a**–**c**), H150 (**d**–**f**), K15 (**g**–**i**), Af380 (**j**–**l**) and Af450 (**m**–**o**), combined with Hoechst’s chromatin staining (pseudocoloured red). Filled and empty arrowheads correspond to transfected and non-transfected cells, respectively. Micrographs are maximum intensity projections of three consecutive optical sections separated by 0.24 μm, obtained by structured illumination microscopy. Scale bar: 20 µm (applies to **a**–**o**)

**Fig. 3:**
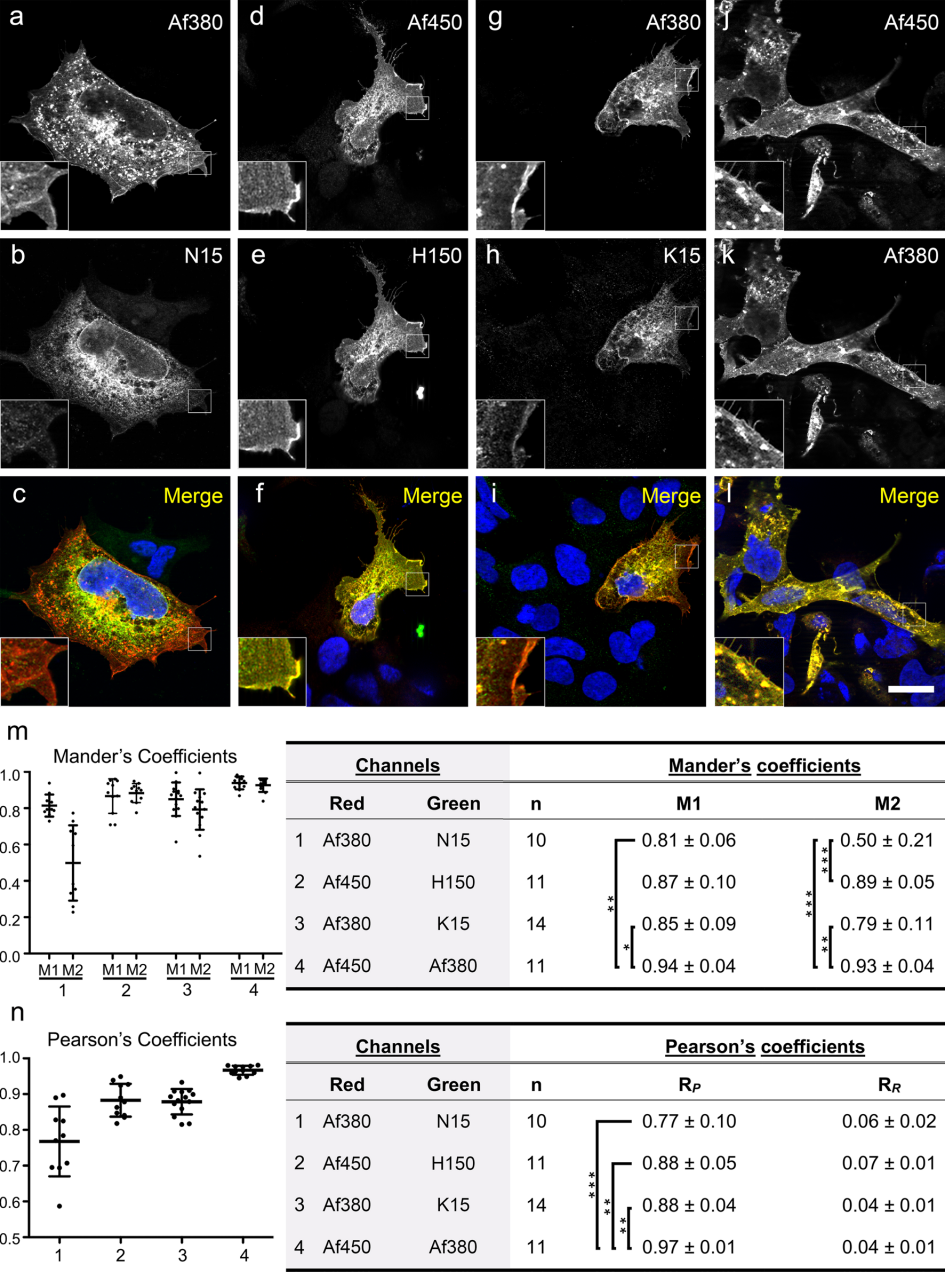
Double immunofluorescence labelling of CB_1_-transfected HEK293 cells (**a**–**l**) with combined goat and rabbit anti-CB_1_ receptor antibody couples: Af380/N15 (**a**–**c**), Af450/ H150 (**d**–**f**), Af380/K15 (**g**–**i**) and Af450/Af380 (**j**–**l**). Single-channel images shown in **a**, **d**, **g**, **j** and in **b**, **e**, **h**, **i** were pseudocoloured red and green, respectively, and together with Hoechst’s chromatin staining to generate images shown in **c**, **f**, **i**, **l**. Scale bar: 20 µm (applies to **a**–**l**). Micrographs are maximum intensity projections of three consecutive optical sections separated by 0.24 μm, obtained by structured illumination microscopy (**m**–**n**). Measurement of the Mander’s pixel overlap *M*1 and *M*2 and Pearson’s *R*_*P*_ pixel intensity correlation coefficients between the two immunofluorescence signals of doubly immunostained cells. Graphs in **m**–**n** show plots and mean ± SD values of the Mander’s and Pearson’s coefficients. Kruskal–Wallis ANOVA yielded statistically significant differences between groups for *M*1 (*P* < 0.005) and *M*2 coefficients (*P* < 0.0001) and *R*_*P*_ (*P* < 0.0001) values. Asterisks in tables refer to significant differences from Dunn’s multiple comparison test (*P < 0.05; **P < 0.01; ***P < 0.001)

The ability of antibodies to specifically bind their target antigen was first tested by immunofluorescence in paraformaldehyde fixed and permeabilized HEK-293 cells transiently transfected with an expression plasmid encoding for the full-length human CB_1_ receptor. Under these conditions, all the five anti-CB_1_ antibodies clearly detected CB_1_-transfected cells, although N15 and K15 antibodies required longer exposure times to capture immunofluorescence signals to the point just below saturation, and N15 antibody produced a diffuse background staining in non-transfected cells. Intense intracellular immunostaining was seen with all five antibodies, whereas plasma membrane staining was more variable (Fig. 2). As a more accurate indicator of the variable ability of the different antibodies to recognize their target antigens in CB_1_ overexpressing HEK-293 cells, we combined anti-CB_1_ antibodies raised in different species for double immunofluorescence in fixed and permeabilized cells. As expected for couples of antibodies against the same target protein, all combinations resulted in double staining of CB_1_ receptor-transfected cells, but with differences in the extent of co-localization. Thus, stainings produced by N15 and Af380 antibodies were highly co-localized in the cytoplasm surrounding the cell nucleus but not in the periphery of the cell, including the plasma membrane, where Af450 antibody produced a considerably stronger immunoreactivity than N15 (Fig. 2a–c). Combination of antibody couples H150/Af450 (Fig. 3d–f) and Af380/Af450 (Fig. 3j–l) led to an almost complete co-localization throughout the entire cell, whereas K15/Af380 combination produced a reddish-coloured plasma membrane in merged images as a consequence of a considerably weaker surface labelling with K15 antibody compared with Af450 (Fig. 3g–i). These findings were quantitatively analysed by measuring the percent pixel overlap (Mander’s *M*1 and *M*2 coefficients) and the pixel intensity correlation (Pearson’s *R*_*P*_ coefficient) between the two immunofluorescence signals of doubly immunostained cells. Kruskal–Wallis ANOVA revealed statistically significant differences for both *M*1 (*P* < 0.005) and *M*2 coefficients (*P* < 0.0001), whereas Dunn’s multiple comparison test found significant differences between selected pairs of antibody combinations (Fig. 3m). Thus, according to qualitative observations, both *M*1 and *M*2 coefficients were close to 1.0 for the Af450/Af380 combination (*M*1, 0.94 ± 0.04 SD, *M*2, 0.93 ± 0.04 SD) and slightly lower, but not statistically different, for the Af450/H150 combination (*M*1, 0.87 ± 0.10 SD; *M*2, 0.89 ± 0.05 SD). Lower Mander’s coefficients, particularly *M*2, were obtained for the Af380/K15 pair (*M*1, 0.85 ± 0.09 SD; *M*2, 0.79 ± 0.11 SD), reaching statistically significant differences compared with the Af450/Af380 pair. As expected, the lowest coefficients were observed for the Af380/N15 combination, with a remarkably low mean value of *M*2 (*M*1, 0.81 ± 0.06 SD; *M*2, 0.50 ± 0.21 SD) (Fig. 3m), consistent with the observation that immunostaining with the Af380 antibody involved a considerably larger cell area than with N15, which was predominantly intracellular (Fig. 3a–c). In consequence, the resulting *M*1 and *M*2 coefficients were statistically lower compared with those obtained for the Af450/Af380 pair, whereas the value of *M*2 was statistically different also for the comparison between the Af380-N15 and Af450-H150 combinations. In addition to yielding the highest Mander’s overlap coefficients when combined, the Af380, Af450 and H150 antibodies produced the most intense immunofluorescence staining. Thus, Af380 showed the shortest exposure time to achieve immunofluorescence signal just below saturation, closely followed by Af450 and H150 antibodies (2.4 ± 1.2 SD and 3.5 ± 1.2 SD fold longer exposure times, respectively). By contrast, much longer exposure times were observed with N15 and K15 antibodies (13.3 ± 4.7 SD and 21.4 ± 6.9 SD fold longer, respectively). Measurement of Pearson’s *R*_*P*_ coefficients revealed a high degree of positive pixel intensity spatial correlation between immunofluorescence signals generated by all couples of antibodies. Coste’s randomization analysis yielded *R*_*R*_ values close to 0 with a confidence limit above 99% for all individual images, showing that Pearson’s *R*_*P*_ coefficients did not originate by random chance (Fig. 3n). Despite the significant positive correlation found in all antibody combinations, there were marked and statistically significant differences in *R*_*P*_ values (Kruskal–Wallis ANOVA, *P* < 0.0001). Thus, the highest and lowest mean *R*_*P*_ values for the co-localization within the dual colour fluorescence images corresponded to the Af450/Af380 and Af380/N15 combinations (0.97 ± 0.01 SD and 0.77 ± 0.10 SD, respectively), whereas Af450/H150 and Af380/K15 combinations yielded very high *R*_*P*_ values (0.88 ± 0.05 SD and 0.88 ± 0.04 SD, respectively), but significantly lower in comparison with Af450/Af380 combination as revealed by Dunn’s multiple comparison test (*P* < 0.01). Again, consistent with qualitative findings, the highest significance was detected between Af450/Af380 and Af380/N15 combinations (*P* < 0.001) (Fig. 3n).

**Fig. 4:**
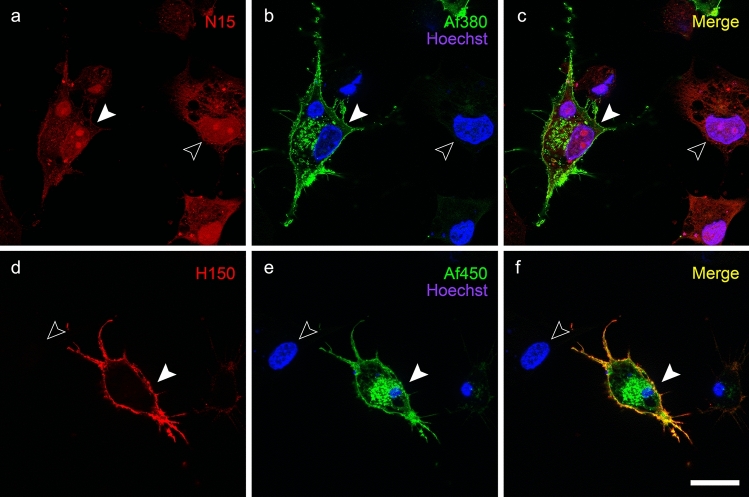
Live immunolabelling of in CB_1_-transfected HEK293 cells with N15 and H150 antibodies (pseudocoloured red), raised against the extracellular amino-terminal tail of CB_1_ receptor, followed by cell fixation–permeabilization and co-immunolabelling with Af380 (**a**–**c**) and Af450 (**d**–**f**) antibodies (pseudocoloured green), raised against the carboxy-terminus of CB_1_ receptor. Nuclei were counterstained using Hoechst nuclear stain. Single-channel images shown in **a**, **b** and **d**, **e** were merged together with Hoechst’s chromatin staining (shown in **c** and **f**, respectively). Micrographs are maximum intensity projections of three consecutive optical sections separated by 0.24 μm, obtained by structured illumination microscopy. Scale bar: 20 µm (applies to **a**–**l**)

To test the ability of anti-CB_1_ receptor N-terminal antibodies to bind their target antigens in native conditions, double immunofluorescence assays were performed by incubation of live cells with either goat N15 or rabbit H150 polyclonal antibodies followed by paraformaldehyde fixation and incubation with rabbit Af380 and goat Af450 antibodies, respectively. In these conditions, plasma membrane staining with N15 antibody was variable among the CB_1_-transfected cell. Thus, N15 antibody detected clear plasma membrane staining after long exposure times in a subset of cells, causing a marked intracellular background autofluorescence to emerge (Fig. S1a–f). On the contrary, faint or no membrane staining was observed using similar acquisition settings in other cells (Fig. 4a–c). By contrast, H150 antibody systematically produced strong plasma membrane immunofluorescence staining, with virtually no background, which was highly co-localized with plasma membrane staining produced by Af450 antibody (Fig. 4d–f). These results indicate that antigen masking caused by paraformaldehyde fixation does not account for the different ability of N15 and H150 antibodies to detect CB_1_ receptors localized at the plasma membrane.

**Fig. 5:**
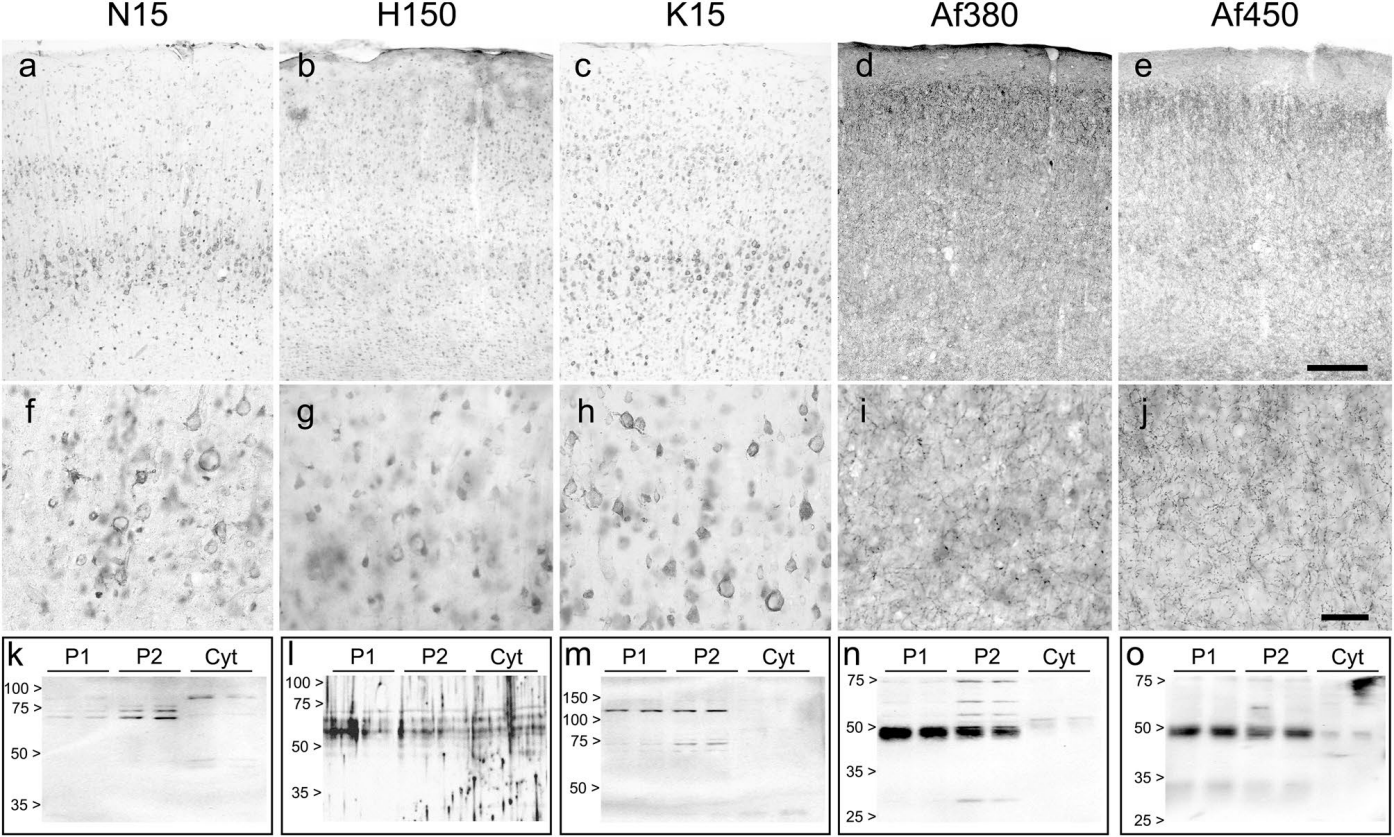
(**a**–**j**) Micrographs of coronal sections of the adult rat parietal cortex immunostained with the different antibodies designed against CB_1_ receptor tested here. (**a**–**e**) Low-magnification micrographs show the overall distribution of immunoreactivity throughout the depth of the cortex using the different antibodies. (**f**–**j**) Higher-magnification micrographs show details of the immunostaining pattern in cortical layer V. Scale bars: 200 µm in **e** (applies to **a**–**e**); 50 µm in **j** (applies to **f**–**j**). (**k**–**o**) Western blot analysis using the different antibodies against the CB_1_ receptor tested in this study. Equivalent amounts of protein (20 µg/lane) from P1, P2 and Cyt fractions obtained from homogenates of adult rat brain cortex were loaded in duplicate and run in parallel

Next, the anti-CB_1_ antibodies were assayed by immunohistochemistry for their ability to specifically bind CB_1_ receptor on histological sections from paraformaldehyde-fixed adult rat brain cortex. Under these conditions, all the five antibodies led to an unevenly distributed immunostaining pattern throughout cortical layers I–VI. However, the dorsoventral distribution of immunolabelling and the types of detected cellular and subcellular structures varied considerably with the different antibodies used (Fig. 5a–j). Goat polyclonal antibodies N15 and K15, raised against the N-terminal region and against a sequence within the cytosolic C-terminal tail of CB_1_ receptor, respectively (Fig. 1), yielded a marked somatic immunostaining throughout cortical layers II–VI that was considerably more intense in layer V than in the rest (Fig. 5a, c). Immunolabelling with these two antibodies clearly delineated neuronal perikarya of variable morphology (Fig. 5f, h). A similar but more diffuse distribution pattern could be observed with the rabbit polyclonal antibody H150 (Fig. 5b, g), which was designed against a large peptide encompassing the extracellular N-terminal tail, the first transmembrane domain and most of the intracellular loop 1 of CB_1_ receptor (Fig. 1). By contrast, the anti-CB_1_ rabbit polyclonal Af380 and goat polyclonal Af450 antibodies, raised in rabbit against the C-terminal 31 amino acids of CB_1_ receptor, produced a neuropil staining throughout the cortical depth, being more intense in layers II/III than in the rest (Fig. 5d, e). As seen at high magnification, this neuropil staining consisted of fibre profiles decorated with intensely stained presynaptic-like boutons (Fig. 5i, j). In summary, only Af380 and Af450 produced an immunostaining pattern consistent with the laminar and subcellular distribution previously described in the rodent brain cortex (Egertová and Elphick 2000; Bodor et al. 2005; Deshmukh et al. 2007).

To test the ability of the different antibodies to recognize denatured CB_1_ receptor from brain tissue, Western blot assays were performed in samples of P1, P2 and Cyt fractions obtained from adult rat brain cortex. Both N15 and K15 antibodies detected several bands migrating slightly below the 70 kDa standard in P1 and P2 fractions, which were more intense in P2 than in P1 fraction (Fig. 5k, m). Additionally, N15 antibody detected a band migrating between the 75 and 100 kDa standards in Cyt fraction (Fig. 5K) and K15 antibody yielded a net band of similar intensity P1 and P2 fractions and migrating above the 100 kDa standard (Fig. 5m). In the three fractions analysed, intense bands were detected above the 50 kDa standard with H150 antibody, with no clear differences in the signal intensity seen among the different fractions (Fig. 5l). Both Af380 and Af450 antibodies detected a major band around the 50 kDa standard in P1 and P2 but not in Cyt fraction (Fig. 5n–o). As a test of the specificity of immunohistochemical and Western blot staining, we performed immnohistochemical and Western blot assays before and after preadsorption of antibodies with the corresponding antigenic peptides, which were available for N15, K15, Af380 and Af450 antibodies, but not H150. Antigen-preabsorbed and non-preabsorbed N15 and K15 antibodies produced a similar pattern of immunohistochemical staining and detected similar bands in Western blot analysis (Fig. S2a, b), indicating that the signals observed resulted from non-specific binding of antibodies. By contrast, both immunohistochemical signals and the major immunoreactive band found in P1 and P2 fractions at ~ 50 kDa with both Af380 and Af450 antibodies were virtually undetectable following preadsorption of the primary antibodies with the specific blocking peptide (Fig. S2a, b). These findings suggested that both the immunohistochemical staining pattern observed and the ~ 50 kDa immunoreactive band detected in P1 fraction and P2 membranes resulted from specific binding of Af380 and Af450 antibodies to CB_1_ receptor.

**Fig. 6:**
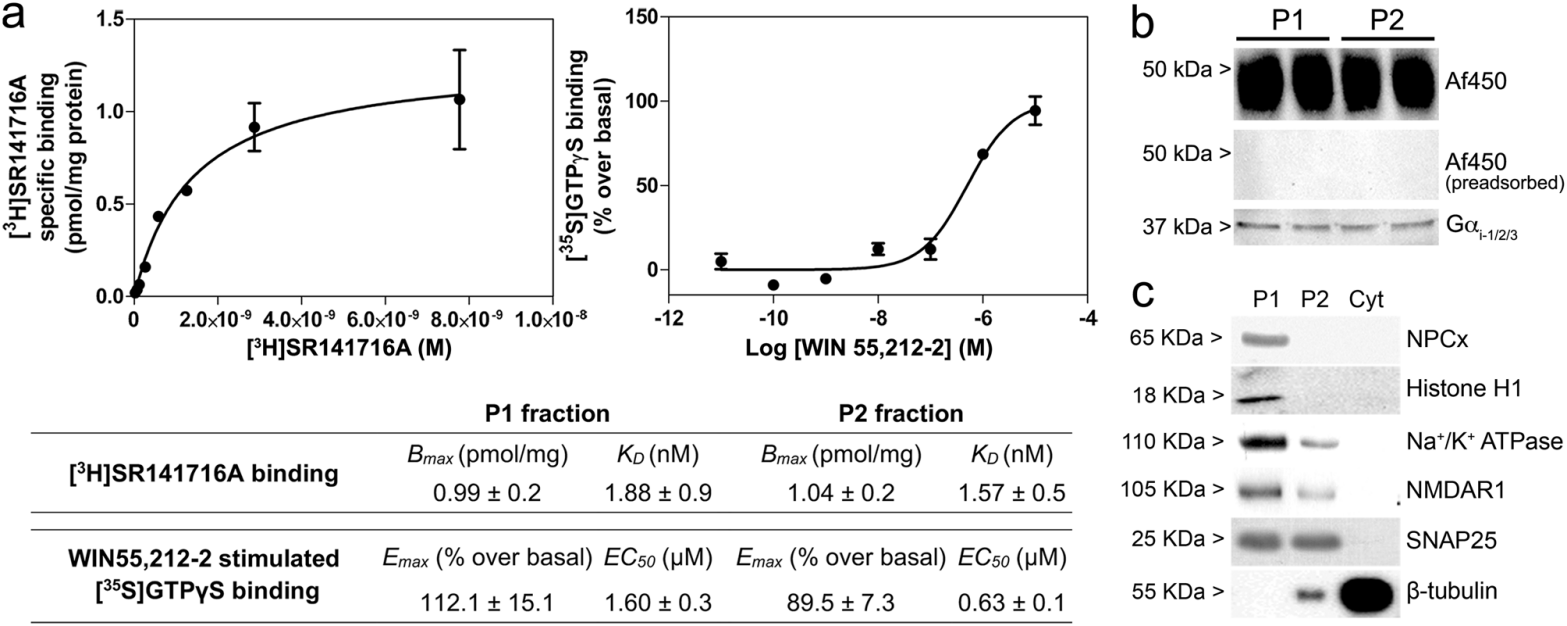
**a** Radioligand and [^35^S]GTPγS binding assays in P1 and P2 fractions from the adult rat brain cortex. Representative saturation binding curve for [^3^H]SR141716A (0.01–10 nM) and representative dose–response curve of WIN 55,212-2 (0.1 nM–10 μM) stimulated [^35^S]GTPγS binding (right), both in P1 fraction from the adult rat cortex. Non-specific binding in stimulated [^35^S]GTPγS binding assays was determined in the presence of 10 μM unlabelled GTPγS. Each point in both curves corresponds to the mean ± SEM value of one representative experiment performed in triplicate and duplicate for radioligand and [^35^S]GTPγS binding assays, respectively. The accompanying table shows maximal number of sites (*B*_max_) and affinity (*K*_*D*_) values for the selective CB_1_ receptor antagonist [^3^H]SR141716A and the maximal effect (*E*_max_) and potency (EC_50_) of the CB_1_ cannabinoid receptor agonist WIN 55,212-2 to stimulate [^35^S]GTPγS binding assays in P1 and P2 subcellular fractions of the adult rat cortex. Values shown in the table are mean ± SEM of at least three independent experiments. **b** Western blot analysis against CB_1_ receptor and G inhibitory protein alpha subunits (*G*α_i_-_1,2,3_) in both P1 and P2 subcellular fractions from adult rat brain cortex (20 µg/lane). **c** Western blot analysis of P1, P2 and Cyt fractions from the adult rat cortex (10 µg/lane) with antibodies against subcellular fraction-specific antigens: NPCx (62 kDa component of the nuclear pore complex), Histone H1, Na^+^/K^+^ ATPase (α_1_ subunit of Na^+^/K^+^ ATPase); NMDAR1 (NR1 subunit of the NMDA receptor), SNAP25 (synaptosome-associated protein 25) and β-tubulin (see Supplementary Table S1 for further details)

The presence of an antigen preadsorption-sensitive ~ 50 kDa strong immunoreactivity band for anti-CB_1_ Af380 and Af450 antibodies in P1 samples (likely enriched in cell nuclei) prompted us to analyse the density of CB_1_ receptor-specific ligand binding sites in P1 fraction and to compare it with that in P2 membranes (known to be enriched in plasma membrane). To this end, we performed saturation binding experiments with the selective CB_1_ receptor radioligand antagonist [^3^H]SR141716A (0.1–10 nM), showing that this compound labelled a single and homogeneous population of binding sites with a similar maximal density in both P1 and P2 samples (Fig. 6a), further demonstrating the presence of CB_1_ receptors in both fractions. Moreover, the CB_1_ receptor agonist WIN 55,212-2 was able to stimulate guanosine-5′-O-(3^−^[^35^S]thio)-triphosphate ([^35^S]GTPγS) binding in both fractions, with a slightly higher efficiency and lower potency in P1 (*E*max = 121.1 ± 015.1% above basal; EC_*50*_ = 0.63 ± 0.06 μM) than in P2 (*E*max = 89.5 ± 7.3% above basal; EC_*50*_ = 1.60 ± 0.3 μM) (Fig. 6a), showing that CB_1_ cannabinoid receptors are efficiently coupled to *G*_i/o_ proteins in both P1 and P2 fractions from the adult rat cerebral cortex. Accordingly, key molecular components of this signal transduction pathway, i.e., *G* inhibitory protein alpha subunits (*G*α_i-1_, *G*α_i-2_ and *G*α_i-3_), could be detected by immunoblot in both fractions by using an antibody raised against a peptide common to three major G inhibitory protein alpha subunits (*G*α_i-1_, *G*α_i-2_ and *G*α_i-3_) (Fig. 6b). To ascertain whether the concurrence of CB_1_-immunoreactivity, CB_1_ receptor-specific ligand binding and CB_1_ receptor agonist-stimulated [^35^S]GTPγS binding in P1 nuclear fractions could be explained by the presence of cell membranes pelleted during the first centrifugation step after tissue homogenization, we performed Western blot experiments in P1, P2 and Cyt fractions using specific markers of subcellular compartments (see Table S1 for details). Although P1 membranes displayed immunoreactivity for NPCx and histone H1 proteins, revealing the enrichment of P1 in cell nuclei, strong immunoreactivity was also observed for the plasma membrane markers Na^+^/K^+^ ATPase, NMDAR1 and SNAP25, whereas no signal was detected for the cytosolic marker β-tubulin (Fig. 6c). Therefore, the concurrence of CB_1_ immunoreactivity, CB_1_ receptor-specific ligand binding sites and CB_1_ receptor coupling to *G*_i/o_ proteins in P1 fractions was very likely due to the presence of cell membranes in these samples.

**Fig. 7:**
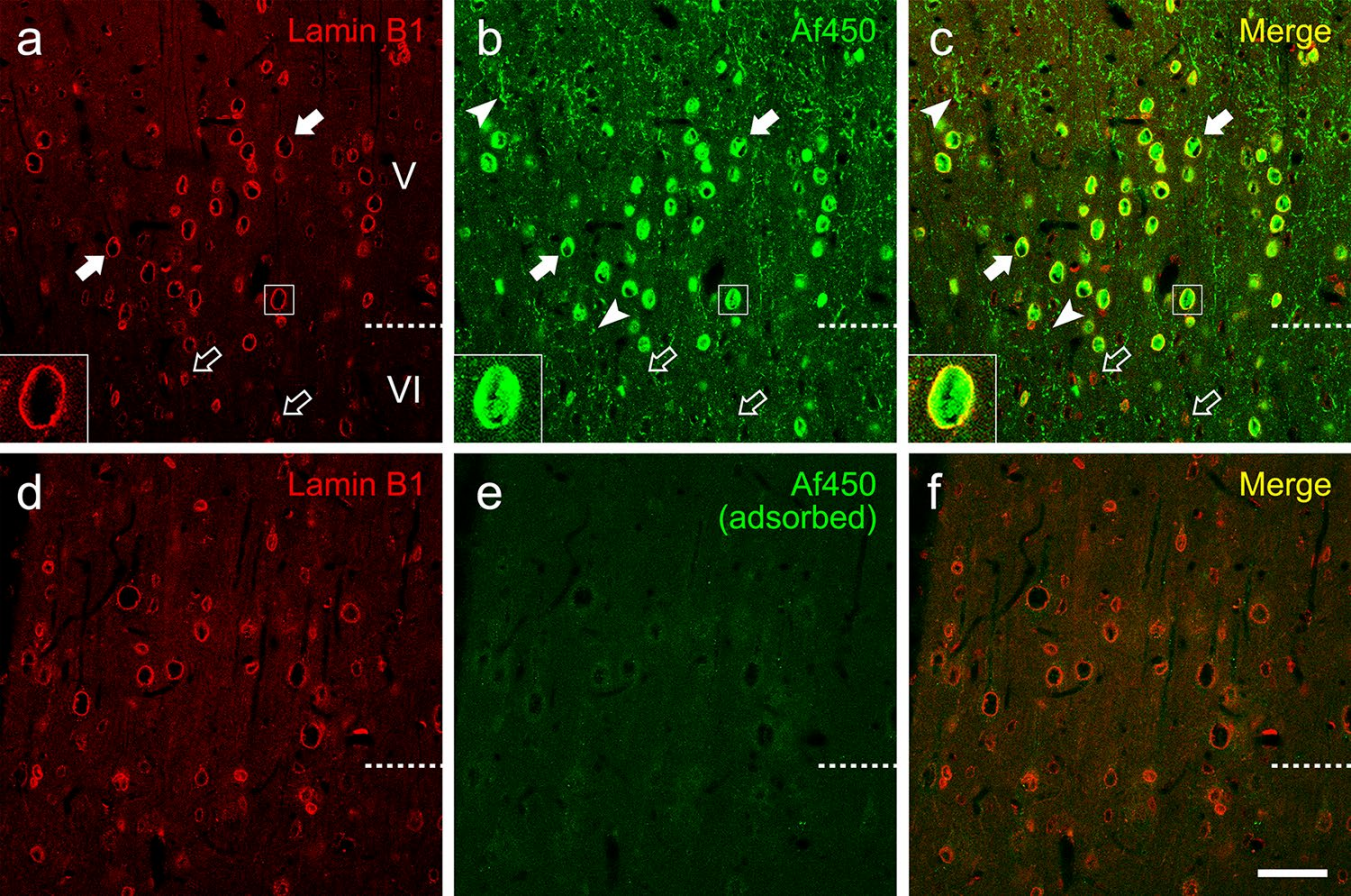
**a**–**c** Double immunofluorescence labelling in the adult rat cortex under sodium sulphide fixation combining a mouse monoclonal antibody recognizing the nuclear protein lamin B1 (pseudocoloured red) and the goat polyclonal anti-CB_1_ antibody Af450 (pseudocoloured green). The anti-CB_1_ antibody immunostained presynaptic-like boutons (arrowheads) as well as large and medium sized (filled arrows) but not small nuclei (empty arrows), as seen by co-staining with lamin B1. **d**–**f** The same experiment depicted in **a**–**c** was performed after preadsorption of the anti-CB_1_ with the immunizing peptide. Images are single 0.5-µm-thick confocal optical sections. Scale bar: 50 µm in **f** (applies to **a**–**f**)

The results shown so far demonstrate that all the five antibodies tested are able to bind CB_1_ receptors in transiently transfected HEK-293 cells, although with variable capacity to detect discrete subcellular pools of CB_1_ receptor (Figs. 2–4, S1). However, only Af380 and Af450 antibodies clearly labelled axons and presynaptic-like boutons in tissue sections of the adult rat cortex, consistent with the expected distribution of CB_1_ receptor (Figs. 5, S2). Therefore, we attempted to rescue (N15, H150 and K15 antibodies) and/or enhance (Af380 and Af450) specific immunostaining with all the five antibodies in sections of adult rat brain cortex, using a fixation method, consisting of a brief perfusion with sodium sulphide buffer before aldehyde fixation, which has been previously shown to improve antibody sensitivity without compromising specificity for a variety of antigens (Mitchell et al. 1993; Montaña et al. 2012; García del Caño et al. 2015). Under these conditions, N15, H150 and K15 antibodies produced a similar pattern as compared with standard conditions, and immunostaining was insensitive to preadsorption of N15 and K15 antibodies with their corresponding blocking peptides. By contrast, under sulphide fixation, Af380 immunostaining intensity increased considerably in axonal profiles and presynaptic-like puncta throughout the depth of the cortex, particularly in layers II/III (Fig. S3 and Supplementary results for further details). Strikingly, in cortical tissue subjected to sodium sulphide fixation, Af450 antibody produced a somatic immunostaining composed of round profiles that resembled cell nuclei, along with the axonal and presynaptic-like immunostaining pattern already observed under standard fixation (Fig. S4 and Supplementary results for further details). Combination of Af450 and anti-lamin B1 antibodies in double immunofluorescence assays revealed the presence of Af450 immunoreactivity in large and medium sized nuclei of the rat cortex (Fig. 7b, c), and this particular labelling pattern was mostly internal to the nuclear lamina (Fig. 7a–c, Insets). Of note, preadsorption of the primary antibody with the specific blocking peptide abolished both presynaptic and nuclear staining produced by Af450 antibody (Fig. 7d–f), showing that sulphide fixation unmasks binding sites of Af450 antibody within the nucleus without affecting the presynaptic staining observed in sections from brain tissue fixed by the standard method.

**Fig. 8:**
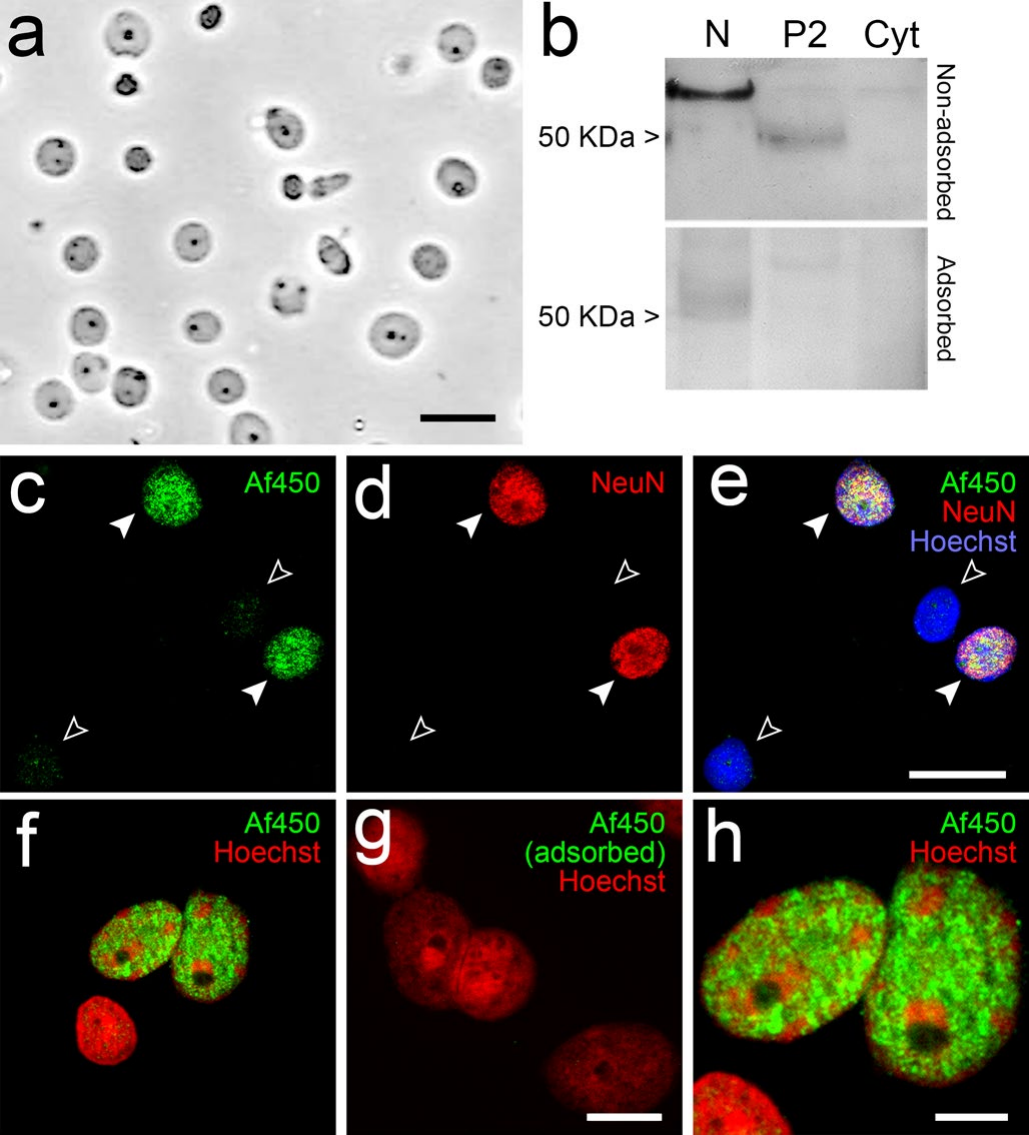
Analysis of immunoreactivity of the goat polyclonal Af450 antibody in intact nuclei (N) isolated from adult rat brain cortex. **a** Representative image of phase-contrast microscopy of nuclei isolated from a homogenate of adult rat cerebral cortex showing no debris or contamination by other organelles. Scale bar: 20 µm. **b** Western blot analysis of N, P2 and Cyt from the adult rat brain cortex using Af450 antibody. Bands of ~ 62 kDa and ~ 50 kDa detected in N and P2 samples, respectively, disappeared after preadsorption of the antibody with the immunizing peptide. Equal amounts of protein (10 µg/lane) were loaded and run in parallel. **c**–**e** Double immunofluorescence labelling with Af450 (pseudocoloured green) and anti-NeuN/Fox-3 (NeuN, pseudocoloured green) antibodies combined with Hoechst’s staining (pseudocoloured blue). The Af450 antibody detected a strong signal in every NeuN-immunopositive nucleus (filled arrowheads), whereas very weak or no staining was observed in nuclei devoid of NeuN/Fox-3 (empty arrowheads). Scale bar: 20 µm in **e** (applies to **c**–**d**). **f**–**h** Combined immunofluorescence with the Af450 antibody (pseudocoloured green) and Hoechst’s chromatin staining (pseudocoloured red) in isolated cell nuclei from the adult rat cortex before (**f**) and after (**g**) incubation of the primary antibody with the specific blocking peptide. The higher-magnification micrograph (**h**) of the nuclei shown in **f** depicts the non-overlapping pattern between Af380 immunofluorescence and patches of intense Hoechst’s staining. Scale bars: 10 µm in **g** (applies to **f**–**g**); 5 µm in **h**

To analyse more in depth the origin of the nuclear signal observed with Af450 antibody, we isolated highly purified intact nuclei (N fraction) from the adult rat cortex (Fig. 8a) for Western blot and immunofluorescence analysis. Immunoblot on N samples using the Af450 antibody yielded a net band at ~ 60 kDa clearly above the theoretical 52 kDa molecular mass of rat CB_1_ receptor observed in P2 samples (Fig. 8b). Consistent with that observed in tissue sections under sulphide fixation, Af450 antibody produced strong immunoreactivity in intact nuclei isolated from the adult rat cortex. Notably, co-immunolabelling with the neuronal marker NeuN/Fox-3 revealed that only neuronal nuclei were Af450 positive (Fig. 8c–e). Again, Af450 immunoreactivity in N samples was virtually abolished by preadsorption of the primary antibody with the immunizing peptide, both in Western blot (Fig. 8b) and immunofluorescence assays (Fig. 8f–g). High-resolution images showed that Af450 immunoreactivity was distributed throughout the nucleoplasm in subdomains poor in chromatin (Fig. 8h, S5a–c), internal to the nuclear lamina (Fig. S5d–f) and partially overlapping with components of the nuclear matrix NeuN/Fox-3 (Fig. S5g–i) and SC35 (Fig. S5j–l), which is inconsistent with the expected localization of an integral membrane protein, which is predicted to partition into cell membranes. These results, pointing strongly to the conclusion that the signal detected by the Af380 antibody in nuclei is non-specific, were confirmed by saturation radioligand binding and agonist-stimulated [^35^S]GTPγS binding assays in N samples, since no CB_1_ receptor-specific sites or agonist-stimulated CB_1_ receptor coupling could be detected (see Fig. S6 for details).

**Fig. 9:**
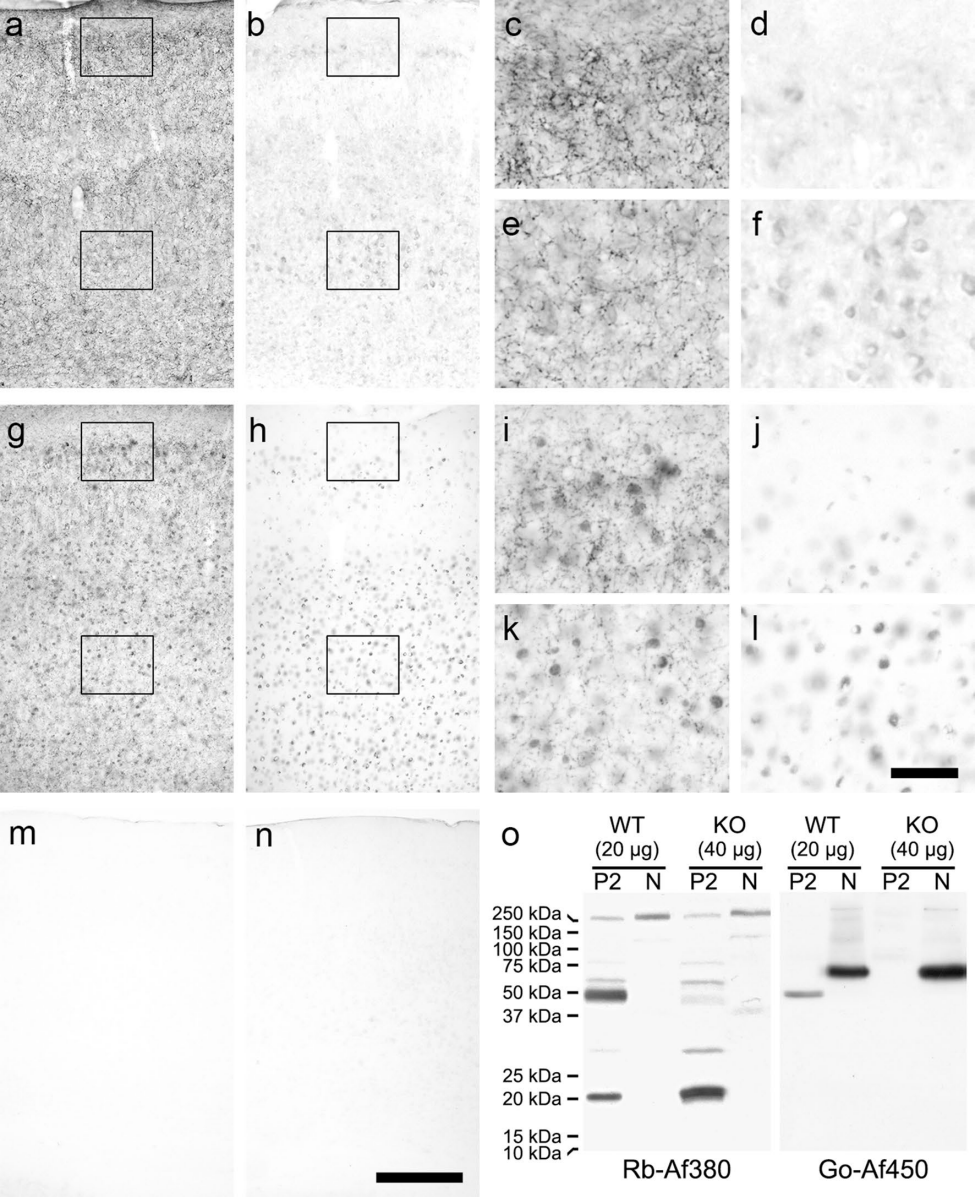
Anti-CB_1_ receptor immunohistochemical staining in sulphide-fixed parietal cortex sections and Western blot analysis of P2 and N fractions from CB_1_-WT and CB_1_-KO mice generated by the Ledent’s lab (Ledent et al. 1999) using the rabbit polyclonal Af380 and the goat polyclonal Af450 antibodies. **a**–**f** Micrographs showing the distribution of immunoreactivity produced by the Af380 antibody in the parietal cortex of CB_1_-WT (**a**, **c**, **e**) and CB_1_-KO (**b**, **d**, **f**) mice. Framed areas in panoramic images **a** and **b** are shown at higher magnification in **c**, **d** and **e**, **f** respectively. **g**–**l** Micrographs showing the distribution of immunoreactivity produced by the Af450 antibody in the parietal cortex of CB_1_-WT (**g**, **i**, **k**) and CB_1_-KO (**h**, **j**, **l**) mice. Framed areas in panoramic images **g** and **h** are shown at higher magnification in **i**–**k** and **j**–**l**, respectively. **m**–**n** Low-magnification micrographs showing the absence of immunostaining in the parietal cortex of CB_1_-WT (**m**) and CB_1_-KO (**n**) when Af450 antibody was used after being preabsorbed with the immunizing peptide. Scale bars: 200 µm in **n** (applies to **a**, **b**, **g**, **h**, **m**, **n**), 50 µm in **l** (applies to **c**–**f**, **i**–**l**). **o** Western blot analysis of P2 and N fractions isolated from the adult mouse brain cortex of Ledent’s CB_1_-WT and CB_1_-KO mice using either the Af380 (left immunoblot) or Go-Af380 (right immunoblot) antibodies. Double amount of protein was loaded from CB_1_-KO mice samples

Regardless of the non-specific binding or Af450 antibody to a nuclear protein, only Af380 and Af450 antibodies passed the specificity tests in all samples and conditions used so far. Therefore, to further analyse CB_1_ receptor-specific and non-specific signals produced by these two antibodies, we performed immunohistochemical and Western blot analyses in CB_1_-WT and CB_1_-KO mice of the Ledent’s line (Ledent et al. 1999). Since, in sections of the rat cerebral cortex, the Af380 antibody produced a stronger immunohistochemical signal under sulphide fixation (compared with the standard method) and sulphide fixation unmasked non-specific binding sites of the Af450 antibody, we used tissue processed under these conditions for immunohistochemistry in CB_1_-WT and CB_1_-KO mice. The immunohistochemical patterns produced by Af380 and Af450 antibodies in sulphide-fixed cerebral cortex sections of CB_1_-WT adult mice were qualitatively indistinguishable from those observed in the adult rat cortex; i.e., Af380 antibody produced a neuropil staining consisting of a network of axonal profiles with a characteristic layered-pattern distribution (Fig. 9a, c, e), whereas Af450 antibody produced the same pattern, along with a strong immunostaining in cell nuclei (Fig. 9g, i, k) as we had observed in the adult rat cortex. In CB_1_-KO mice, immunostained sections of CB_1_-KO mice with either of the two antibodies were completely devoid of fibres and presynaptic-like puncta (Fig. 9b, d, f, h, j, l). However, the Af380 antibody produced a pale but clearly prominent immunostaining against the surrounding unlabelled neuropil in numerous cell bodies (Fig. 9b, d, f), whereas the Af450 antibody stained numerous cell nuclei (Fig. 9h, j, l) consistent with a non-specific nature of nuclear staining. Non-specific signals produced by both antibodies were particularly prominent in layer V. Before carrying out Western blot analysis on isolated subcellular fractions from CB_1_-WT and CB_1_-KO mice, samples from both genotypes were analysed by CB_1_ receptor agonist-induced [^35^S]GTPγS binding and by PCR amplification, respectively. As expected, the CB_1_ receptor agonist WIN 55,212–2 was able to stimulate [^35^S]GTPγS binding in P2 samples from CB-WT but not CB_1_-KO mice (Fig. S7). Likewise, PCR amplification in cDNA samples obtained by reverse transcription of total RNA (Fig. S8) (isolated from a small piece of the cortical tissue used for Western blotting of intact nuclei) yielded specific PCR products with the three primer pairs used in CB_1_-WT but not CB_1_-KO mice (Fig. S9). Because the strategy to generate Ledent’s CB_1_-KO mouse line led to a null allele that still contains the triplets of Cnr1 gene coding for amino acids 235–473 (Ledent et al. 1999) (Fig. S9a), results of PCR amplification also ruled out the possibility that a transcript containing the coding sequence for the immunizing peptide (residues 443–473 of mouse CB_1_ receptor) could be still expressed in CB_1_-KO mice, thus hindering the interpretation of non-specific signals observed with Af380 and Af450 (Fig. S9 and Supplementary results for further details). Western blot analysis of P2 membranes from CB_1_-WT and CB_1_-KO mice showed that Af380 antibody produced a major band at ~ 50 kDa, which was absent in P2 samples from CB_1_-KO mice, and a second, less intense but clearly positive band around the 20 kDa standard, which still remained in samples from CB_1_-KO mice. By contrast, a single weak band was observed in N samples, from both CB_1_-WT and CB_1_-KO mice, immunoblotted with Af380 antibody. As observed in P2 samples from the adult rat brain, the Af450 antibody produced a single specific band at ~ 50 kDa only in P2 fractions from CB_1_-WT but not CB_1_-KO animals, whereas it detected a single intense band at ~ 60 kDa in N samples obtained from either phenotype (Fig. 9o). Overall, these results show that Af380 and Af450 antibodies bind, both in tissue sections and in denatured samples resolved by SDS-PAGE, not only to the CB_1_ receptor but also to non-CB_1_ receptor targets.

To confirm immunohistochemical results obtained in Ledent’s CB_1_-WT and CB_1_-KO mice, Af380 and Af450 antibodies were assayed in brain sections of CB_1_-WT and CB_1_-KO animals kindly provided by Dr. Giovanni Marsicano (Marsicano et al. 2002), which were generated using a Cre/loxP-based gene-targeting strategy that involves removal of the entire Cnr1 coding region. The immunohistochemical patterns produced by Af380 and Af450 in sulphide-fixed cerebral cortical sections of CB-1WT and CB_1_-KO littermates from this line were virtually identical to those observed in Ledent's model (Fig. S10).

## Discussion

It is well known that the ability of antibodies to recognize their target antigen is highly influenced by the experimental context in which they are used and, therefore, not all antibodies are suitable for all end-use applications (Bordeaux et al. 2010; Voskuil 2014, 2017; Uhlen et al. 2016). The analysis performed here focused on the ability of five anti-CB_1_ receptor antibodies of commercial source, designed against the N-terminal (N15 and H150) or C-terminal (K15, Af380 and Af450) regions of the CB_1_ receptor, to specifically recognize their target in cells transfected with plasmids coding for the native human CB_1_ receptor, in fixed sections from rat and mouse cerebral cortex and in subcellular fractions obtained from rat and mouse brain cortex homogenates. In addition, N15 and H150 antibodies were tested for their ability to detect surface CB_1_ receptor in live cells.

In HEK-293 cells fixed with paraformaldehyde, all the five antibodies tested made it possible to readily distinguish cells transfected with the CB_1_ receptor from non-transfected ones. However, whereas the N-terminal H150 antibody and the C-terminal Af380 and Af450 antibodies produced bright signals that were highly co-localized with each other both intracellularly and on the plasma membrane, the N-terminal N15 and C-terminal K15 antibodies barely and weakly detected surface receptors, respectively. Noteworthy, the H150 antibody was also highly specific and sensitive in detecting surface CB_1_ receptors in live cells, which contrasts with the uselessness of the N15 antibody for this purpose. The distinct peptides used as antigen to generate N15 and H150 antibodies could explain this difference. Indeed, the N15 antibody was raised against an unspecified short sequence near the end of the N-terminal extracellular tail of the human CB_1_ receptor, whereas the H150 antibody was raised against a long peptide spanning residues 1–150 of the human CB_1_ receptor and, therefore, likely to contain more immunogenic sequences. However, this would only explain the differences in sensitivity but not the different ability to detect the surface receptor. Interestingly, it has been demonstrated that a large fraction of CB_1_ receptor are truncated at their N-terminal at early stages in the secretory pathway just before being translocated to the endoplasmic reticulum (ER) (Nordström and Andersson 2006), whereas the untruncated receptors appear to be inefficiently translocated across the ER membrane, leading to high levels of misfolded receptor that are subsequently degraded (Andersson et al. 2003). Although the extent of this truncation has not yet been determined, endogenous truncation of N-terminally c-myc-tagged CB_1_ receptors overexpressed in baby hamster kidney cells has been estimated to cause a mobility shift of about 4 kDa on SDS-PAGE compared with non-truncated ones (Nordström and Andersson 2006). Therefore, considering the 1.2 kDa size of the c-myc tag, it is expected that CB_1_ receptors are cleaved at around residue 26 of the CB_1_ N-tail, probably resulting in the deletion of the N15 antibody epitope. Consequently, the N15 antibody would primarily recognize the newly synthesized N-terminal tail of CB_1_ receptors prior to their translocation to the ER membrane via the secretory pathway used by most GPCRs lacking a cleavable signal peptide, with the first transmembrane domain of the mature receptor functioning as a signal anchor sequence (Wallin and Von Heijne 1995). In addition, N15 antibody could recognize incorrectly folded untruncated CB_1_ receptors retained and subjected to quality control in the ER as well as untruncated CB_1_ receptors that had passed ER quality control (Nordström and Andersson 2006). This is consistent with the observed immunostaining pattern of the N15 antibody, which was distributed mainly around the cell nucleus and showed a weak variable or no staining in the periphery and the plasma membrane of the transfected HEK-293 cells. The fact that the untruncated CB_1_ receptor probably represents only a small fraction of the entire population (Nordström and Andersson 2006) could explain the apparent low sensitivity of the N15 antibody, which in combination with markers of intracellular organelles could be very useful to study specific intracellular species of CB_1_ receptors. Indeed, retained GPCRs have been shown to display enhanced interaction with the ER luminal chaperone BiP, as the primary regulator of endoplasmic reticulum translocation, as well as with carbohydrate-binding chaperones, which are key elements for recycling misfolded receptors through N-linked glycan recognition and processing (Moremen and Molinari 2006; Achour et al. 2008).

When tested on histological sections from the rat cerebral cortex, neither of the two N-terminal antibodies produced a distribution pattern of CB_1_ receptor in fibres and presynaptic-like varicosities widely reported in previous studies (Egertová and Elphick 2000; Bodor et al. 2005; Deshmukh et al. 2007). Although this could be expected for the N15 antibody, it was surprising for the H150 antibody in view of the high specificity and sensitivity that it showed for detecting the CB_1_ receptor in HEK-293 cells. This contrasts with results obtained by other authors showing the ability of antibodies against large fragments of the amino end of CB_1_ receptor (Tsou et al. 1998; Eggan and Lewis 2007) or against a short 15-amino-acid peptide far from the amino end of CB_1_ receptor (Dove Pettit et al. 1998), but not antibodies against a short 14-amino-acid peptide mapping at the extreme end of the N-terminus (Mukhopadhyay and Howlett 2001; Matias et al. 2002), to produce immunohistochemical signals in brain tissue sections consistent with the accepted gross anatomical and fine distribution patterns for the CB_1_ receptor (Dove Pettit et al. 1998; Tsou et al. 1998; Eggan and Lewis 2007). Intriguingly, in one of the few studies devoted to the analysis of the specificity of anti-CB_1_ antibodies (Grimsey et al. 2008), authors reported that two commercial antibodies produced against residues 1–77 (PA1-745, Affinity BioReagents) and 1–99 (C1108, Sigma-Aldrich) of the rat and human CB_1_ receptors, respectively, failed to detect the CB_1_ receptor in histological sections of rodent brain, not even in HEK-293 cells transfected with a HA-tagged human CB_1_ receptor, and neither in live cells nor in paraformaldehyde-fixed and permeabilized cells. In agreement with these negative results, other commercial antibody against rat CB_1_ receptor N-terminal amino acids 1–77 (C1233, Sigma-Aldrich) and 1–99 (C1108, Sigma-Aldrich) produced immunohistochemical patterns in the cortex, striatum and hippocampus (Fusco et al. 2004) that were inconsistent with the widely accepted distribution of CB_1_ receptor. Differences in immunization and/or purification procedures relative to similar “homemade” antibodies could explain these discrepancies. Coming back to our present data, a possible reason for the lack of specificity of the H150 antibody in rat brain tissue sections could be that this antibody was generated using an immunogen corresponding to a sequence of human origin, since there are eight amino acid discrepancies between the human and rat CB_1_ receptor within a portion of the extracellular N-tail encompassing residues 68–110 and 68–111 of human and rat proteins, respectively. A second possible explanation is that N-linked glycans at asparagine residues 78 and 84 of the rat CB_1_ receptor (Song and Howlet 1995) could affect the recognition of the epitope by the H150 antibody, although this is unlikely since N-glycosylation should have also affected epitope recognition in HEK-293 cells transfected with the human CB_1_ receptor, which is known to be extensively N-glycosylated (Nordström and Andersson 2006; Ruehle et al. 2017).

Again, N15 and H150 antibodies did not detect CB_1_ receptors under denaturing conditions by SDS-PAGE and immunoblotting, as concluded from the analysis of the molecular mass of the immunoreactive bands (both antibodies), antibody–antigen preadsorption control (N15) and the presence of immunoreactive bands of identical size in the fractions of the rat cerebral showing (P1 and P2) and lacking (Cyt) CB_1_ receptor-specific ligand binding sites and CB_1_ receptor coupling to *G*_i/o_ proteins. Similar to what has been discussed about the inability of these N-terminal antibodies to detect the brain CB_1_ receptor by immunohistochemistry, the uselessness of N15 and H150 for Western blotting could also be due to the constitutive N-terminal truncation of the CB_1_ receptor and to differences in the primary sequence between human *versus* rat CB_1_ receptor, respectively. Another possibility is that the large size of the immunogen used to generate the H150 antibody could contain conformational antigenic determinants that would no longer be detectable after denaturation.

Of the three C-terminal antibodies analysed here, the Af450 and Af380 antibodies, raised against 31 amino acids at the extreme carboxy-terminus of mouse CB_1_ receptor were by far the ones that provided the best results in all the final applications tested, although they were not exempt from some issues related to sample processing and end-use application. Double immunofluorescence assays demonstrated a virtual complete co-localization between Af450, and Af380-immunoreactivities, but more importantly, both antibodies produced intense immunoreactivity largely restricted to axonal fibres and presynaptic-like puncta with an excellent signal-to-noise relationship in rat brain cortical sections, and the signals were virtually abolished by peptide preadsorption. This is consistent with several studies that showed a similar CB_1_ immunoreactivity distribution profile using antibodies raised against C-terminal end fragments of variable length, including peptides comprising the last 13 (Egertová and Elphick 2000), 15 (Bodor et al. 2005; Deshmukh et al. 2007; Eggan and Lewis 2007; Eggan et al. 2010) and 73 (Hájos et al. 2000; Wager-Miller et al. 2002; Harkany et al. 2003; Monory et al. 2006; Eggan and Lewis 2007) C-terminal end residues, as well as a number of reports using Af380 (Lafourcade et al. 2007; Yoneda et al. 2013; Diniz et al. 2019; Fuerte-Hortigón et al. 2021) and Af450 (Yoneda et al. 2013; Rivera et al. 2015; Exposito-Alonso et al. 2020) antibodies in the rodent brain cortex. Moreover, both Af380 (Mateo et al. 2017) and Af450 (Lafourcade et al. 2007; Peñasco et al. 2020; Egaña-Huguet et al. 2021) antibodies have proven to be adequate to describe the ultrastructural distribution of CB_1_ receptors and have been validated for specificity in transgenic mice lacking CB_1_ receptor (Hebert-Chatelain et al. 2014b; Remmers et al. 2017; Gutiérrez-Rodríguez et al. 2018). Remarkably, it has been observed that preadsorption of an antibody generated against the 73 C-terminal residues of the CB_1_ receptor with a peptide that spans only the last 15 amino acids removed most but not all of the CB_1_-specific axonal labelling, indicating that the 15 residues at the C-terminus of CB_1_ receptor contained most of the antigenic determinants (Eggan and Lewis 2007). This could explain why the K15 antibody against an unspecified sequence upstream of the last 15 residues was able to recognize the CB_1_ receptor under overexpression conditions in HEK-293, although with a low sensitivity that may not be sufficient to detect physiological levels in brain tissue.

It is well known that the fixation procedure used affects immunohistochemical staining, increasing or reducing specific and non-specific signals or masking antigens to a greater or lesser extent (Fritschy 2008). For instance, Egertová and colleagues reported that the use of Bouin’s fixative for immunohistochemical staining of rodent brain sections with C-terminally directed anti-CB_1_ antibodies abolishes the background staining that still remains when immunogen-preadsorbed antibodies were used in 4% paraformaldehyde-fixed tissue (Egertová et al. 1998; Egertová and Elphick 2000) and preserves only specific immunostaining produced by the same antibodies as seen by comparing tissue sections from CB_1_-WT and CB_1_-KO mice (Egertová et al. 2003; Monory et al. 2006). In addition, certain fixation procedures can prevent epitope masking that occurs as a result of conformational changes in antigens caused by paraformaldehyde fixation. Here, we examined the immunohistochemical staining profile obtained with the different antibodies in histological sections of the adult rat cortex obtained from sulphide-fixed brains. Although this procedure was first developed to reveal zinc-rich terminals using Timm’s staining method (Hassler and Söremark 1968; Sloviter 1982) and, more particularly, as a marker of hippocampal mossy fibre sprouting in animal models of epilepsy (Nadler et al. 1980) and human epilepsy (Sutula et al. 1989), it has been described as a good method to improve immunoreactivity without compromising the immunostaining profile of different proteins (Mitchell et al. 1993); compared with other antigen-retrieval methods, it offers an important advantage for our particular purposes. Specifically, we have previously shown that sulphide fixation improves immunofluorescence signal intensity for phospholipase C beta 1 (PLCβ1) (Montaña et al. 2012) and diacylglycerol lipase α (DAGLα) (García del Caño et al. 2015) and allows their detection in subcellular compartments where they do not appear stained or do so very weakly after standard fixation. Moreover, sulphide fixation did not affect the immunostaining profile of various GABAergic neuronal phenotype markers and glial markers (Montaña et al. 2012) and even increased the immunostaining intensity of subcellular markers such as lamin B1 (García del Caño et al. 2015). Thus, enhancement or maintenance of CB_1_ receptor immunoreactivity after sulphide fixation could allow simultaneous immunostaining of CB_1_ with key components of the brain endocannabinoid system such as PLCβ1 and DAGLα, which are key enzymes for on-demand the synthesis (Shonesy et al. 2015) of the most abundant endogenous CB_1_ agonist (Stella et al. 1997) 2-arachidonoylglycerol (2-AG), as well as with phenotype and subcellular markers. Sulphide fixation did not rescue CB_1_-specific immunolabelling with N15, H150 and K15 antibodies, whereas it increased CB_1_-immunoreactivity with Af380 antibody and led to the emergence of a nuclear staining with Af450 antibody (which was sensitive to peptide preadsorption) with no changes in typical axonal labelling. Interestingly, double immunofluorescence assays in intact nuclei (N fraction) isolated from rat cerebral cortex lacking plasma membrane protein contaminants revealed that the nuclear staining produced by anti-CB_1_ antibody overlapped with components of the nuclear matrix, similar to that previously observed for PLCβ1 and DAGLα (Montaña et al. 2012; García del Caño et al. 2014, 2015). In addition, Western blot analysis on isolated intact nuclei from rat cerebral cortex produced a clean and strong band at ~ 60 kDa that was sensitive to preadsorption of the antibody with the immunogen. Although this signal was clearly above the theoretical 52 kDa molecular mass of the rat CB_1_ receptor, suggesting that it was not related to the CB_1_ receptor, other authors have interpreted the slower migration of these bands on SDS-PAGE as a consequence of N-linked glycosylation at the N-terminal region of CB_1_ receptor (Song and Howlett 1995; Egertová and Elphick 2000; De Jesús et al. 2006). Because such post-translational modifications could lead to a slower mobility of CB_1_ receptors on SDS-PAGE, our findings in intact nuclei still left open the unlikely but feasible and attractive possibility that the signal found in nuclei of neuronal cells with the Af450 antibody could be specific. However, neither specific CB_1_ receptor binding sites nor CB_1_ receptor coupling to inhibitory G proteins could be detected in intact nuclei samples by saturation radioligand binding assays and agonist-stimulated [^35^S]GTPγS binding. In view of these surprising and apparently contradictory results, we performed immunohistochemical assays on cerebral cortex sections of sulphide-fixed brains from a CB_1_-KO mouse line (Ledent et al., 1999). As expected, the Af450 antibody produced the typical fibre staining and presynaptic-like profiles in tissue sections from CB_1_-WT mice but not from CB_1_-KO littermates, whereas a strong nuclear signal was observed in both. Furthermore, Western blot analysis on N samples from CB_1_-WT and CB_1_-KO mice showed that Af450 detected the strong ~ 60 kDa band mentioned above in both genotypes, virtually demonstrating that the nuclear staining produced by the Af450 antibody was not related to the CB_1_ receptor. It is likely that the strong immunoreactivity in cell nuclei observed with the Af450 antibody in both sulphide-fixed tissue sections and N samples resolved by SDS-PAGE results from the binding of IgGs to a sequence of the same CB_1_ receptor-unrelated protein. If that was the case, the observed artefact, rather than be caused by the appearance of “false epitopes” as a consequence of chemical modifications due to sulphide fixation, would be produced by an unmasking effect of a site inaccessible in tissue fixed under standard conditions but for which the Af450 antibody has high affinity when the protein is denatured for Western blot, thus reinforcing our previous evidence showing that sulphide fixation is a good antigen-retrieval method (Montaña et al. 2012; García del Caño et al. 2015) for immunohistochemistry and, furthermore, compatible with electron microscopy (Danscher 1981). Specificity tests with the Af380 antibody in tissue sections of CB_1_-WT and CB_1_-KO littermates subjected to sulphide fixation revealed that, as we had observed in rat tissue sections, this antibody is highly sensitive and specific under these conditions and produced only negligible background staining in brain cortex sections of CB_1_-KO mice, which makes it the best option for use in combination with the immunostaining of antigens that are retrieved by sulphide fixation.

Transgenic animals in which the antigen of interest have been eliminated are considered to be the gold standard for validation of antibody specificity in immunohistochemistry (Saper and Sawchenko 2003; Saper 2005; Bordeaux et al. 2010). However, these models are not free from pitfalls, as exemplified in a recent study on the specificity of four antibodies raised against synthetic peptide sequences corresponding to different parts of the murine CB_2_ cannabinoid receptor (Zhang et al. 2019). In this study, two different CB_2_-KO models generated by homologous recombination that replaced genetic sequences near the 3′ (Zimmer et al. 1999) and 5′ ends (CB_2_-KO strain from Deltagen Inc.) of mouse *Cnr2* exon 3 (which contains the entire mouse CB_2_ receptor encoding region) with the neomycin gene were used. Results of this study indicated that the undeleted *Cnr2* gene sequences still encodes mutant or truncated CB_2_ receptor proteins or fragments that could be detected by anti-CB_2_ antibodies. Of note, the strategy to generate the Ledent’s CB_1_-KO line led to null allele that still contains the triplets of *Cnr1* gene coding for amino acids 235–473 (Ledent et al. 1999). Although Ledent’s mice have been well characterized for the correct transgene insertion and on the basis of a variety of responses to drugs, functional responses and behavioural tests, the evidence in CB_2_-KO models discussed above makes feasible the possibility that a transcript containing the coding sequence for the immunizing peptides for Af380 and Af450 antibodies (residues 443–473 of mouse CB_1_ receptor) could be still expressed in these animals. However, three primer pairs targeting either the deleted or the remaining sequences of the *Cnr1* gene in the Ledent’s CB_1_-KO mouse were unable to amplify any specific mRNA in CB_1_-KO samples by RT-PCR, whereas all the three probes detected CB_1_-specific mRNA sequences in CB_1_-WT littermates. To our knowledge, this is the first time that the possibility that transcripts containing sequences encoding anti-CB_1_ antibody epitopes may still be expressed in Ledent’s CB_1_-KO mice has been addressed. Finally, immunohistochemical staining in histological sections of the cerebral cortex of CB_1_-KO mice in which the entire coding region of the *Cnr1* gene was eliminated (Marsicano et al. 2002) cleared up any doubt, since the Af380 and Af450 antibodies produced specific and non-specific signals identical to those observed in Ledent’s CB_1_-KO mice, ruling out any possibility that the nuclear staining observed in Ledent’s CB_1_-KO mice could be due to the presence of CB_1_ receptor protein fragments.

Of the three C-terminal antibodies used, the K15 antibody failed to detect CB_1_ receptor-specific signals by Western blot, since it produced immunoreactive bands in the P1 and P2 fractions that migrated clearly above the theoretical 52 kDa molecular mass of rat CB_1_ receptor, which in addition were not eliminated by preadsorption of antibodies with an excess of the immunizing peptide. As discussed in relation to the futility of this antibody for immunohistochemical detection of the CB_1_ receptor, the inability of the K15 antibody to detect CB_1_ under denaturing conditions could be due to the fact that most of the antigenic determinants of the C-terminal end of the CB_1_ receptor are located downstream of the antigen used. By contrast, both Af380 and Af450 antibodies were shown to detect a major band migrating slightly below the theoretical 52 kDa molecular mass of rat CB_1_ only in P1 and P2, which contained CB_1_ receptor-specific binding sites and displayed agonist-stimulated CB_1_ receptor coupling to *G*_i/o_ proteins as shown by saturation radioligand binding and agonist-stimulated [^35^S]GTPγS binding assays and, in addition, this ~ 50 kDa band was undetectable when the antibodies were preabsorbed with the immunizing peptide. Af380 antibody detected several other, weaker but clear bands with considerably higher and lower molecular weight in P1 and P2 fractions, whereas extra bands were faint or undetectable with Af450 antibody. The molecular weight of the ~ 50 kDa specific band detected by Western blot in our study agrees with previous reports using the same rabbit Af380 (Fukudome et al. 2004; Yoneda et al. 2013; Rodríguez-Cueto et al. 2016) and goat polyclonal Af450 (Yoneda et al. 2013) antibodies, including two studies from our lab (Peñasco et al. 2020; Egaña-Huguet et al. 2021). Despite the consistency of our data, results obtained by other authors reporting the detection of a major band of about 60 kDa or higher using different anti-CB_1_ receptor antibodies deserve consideration (Song and Howlett 1995; Egertová and Elphick 2000; Mukhopadhyay and Howlett 2001; Wager-Miller et al. 2002; De Jesús et al. 2006; Diniz et al. 2019). Although this slower mobility has been explained as a result of glycosylation of the CB_1_ terminal tail (Song and Howlett 1995; Egertová and Elphick 2000; De Jesús et al. 2006), deglycosylation experiments cause also a considerably mobility shift of the ~ 50 kDa detected with antibodies against large fragments of the N-terminal region or the C-terminal end of CB_1_ receptor (Nordström and Andersson 2006; Esteban et al. 2020), and therefore, these discrepancies must be due to other factors. On the one hand, the antibodies used in some cases were raised against a small fragment of the amino terminal end of the CB_1_ receptor (Song and Howlett 1995; Mukhopadhyay and Howlett 2001), and as discussed extensively above, it is possible that truncation of a short N-terminal fragment of the CB_1_ receptor in the early stages of maturation could cause these antibodies to recognize only untruncated CB_1_ receptors of higher molecular weight, which appear to represent a small fraction of the total receptor population (Nordström and Andersson 2006). On the other hand, heating samples at high temperature causes CB_1_ receptor aggregates with considerably lower mobility in SDS-PAGE (Wager-Miller and Mackie 2016), and it has been recently reported that temperatures above 65 °C favour the formation of high-molecular-weight aggregates (Esteban et al. 2020), which could explain the high-molecular-weight bands migrating considerably above 60 kDa described before (Matias et al. 2002; Wager-Miller et al. 2002). Among the studies focused on the specificity of anti-CB_1_ antibodies intended for Western blotting, it is worth highlighting a recent work devoted to the analysis of the mobility of the CB_1_ receptor on SDS-PAGE using different N-terminal and C-terminal antibodies and CB_1_-KO mice as negative control (Esteban et al. 2020). Noteworthily, only one of the three antibodies used by these authors designed against the first 14 residues of the N-terminal end of the CB_1_ receptor recognized a single CB_1_ receptor-specific band at ~ 64 kDa, whereas two antibodies generated against the C-terminal end of the CB_1_ receptor and one against residues 84–99 of the N-terminal tail (and, therefore, far from the amino end of CB_1_ receptor) detected a single specific band at ~ 50 kDa. Although the authors proposed an interpretation based on the folding and packing state of the protein and the detergent used, their data could be also interpreted as a result of the ability of antibodies against the N-terminal end of the CB_1_ receptor to recognize solely a minor fraction of high-molecular-weight untruncated CB_1_ receptors, which would be below the detection threshold when antibodies are designed against peptides corresponding to sequences located far from the N-terminal extreme. In favour of this interpretation, two of the three antibodies that recognized a specific band of ~ 50 kDa in mouse cerebral cortex and cerebellum could not detect the band of ~ 64 kDa in mouse brain immunoprecipitates prepared with the antibody against the end of the N-tail of CB_1_ receptor, whereas they detected the ~ 50 kDa band in immunoprecipitates prepared with the other two antibodies, probably due to the extremely low density of the putatively untruncated ~ 64 kDa CB_1_ receptors compared to ~ 50 kDa species (Esteban et al. 2020). On the contrary, the antibody against the N-terminal end of the CB_1_ receptor produced a single band at ~ 64 kDa of the same intensity regardless of the antibody used for immunoprecipitation, strongly suggesting that this antibody detects with high sensitivity a minor species of the untruncated receptor. In any case, the results described by Esteban and colleagues (2020) using anti-CB_1_ antibodies designed either against the C-terminus or against residues 84–99 of the N-tail of the CB_1_ receptor are in good agreement with our finding that both Af380 and Af450 recognize a unique CB_1_ receptor-specific band migrating at ~ 50 kDa on SDS-PAGE as seen by analysing immunoreactive bands in P2 membranes from the cerebral cortex of CB_1_-WT and CB_1_-KO littermates. The use of CB_1_-KO animals as negative controls also allowed us to establish Af450 antibody as the best choice for Western blot assays in cortical P2 membranes, since Af380 antibody produced a number of non-specific bands of variable intensity and migrating above and below the ~ 50 kDa CB_1_ receptor-specific band, whereas Af450 led to a single ~ 50 kDa specific band.

In conclusion, of the five antibodies tested for specificity, three of them were identified as the best choice for specific end-use applications. Thus, the ability of the H150 antibody to detect surface receptors in HEK-293 cells overexpressing CB_1_ receptors makes it an excellent unique tool of particular interest to study the dynamics of processes such as internalization, recycling or trafficking to the plasma membrane of CB_1_ receptors. Antibodies Af380 and Af450 provided excellent results for immunodetection of CB_1_ receptors in permeabilized HEK-293 cells and in fixed brain tissue, although only the Af380 antibody is suitable under sulphide fixation when this method is used to increase immunoreactivity for other antigens such as components of the endocannabinoid system or cell phenotype markers. Both Af380 and Af450 antibodies are useful for detection of CB_1_ receptor by Western blot, although the Af380 antibody detects several non-specific bands while Af450 does not, making the latter option the best for Western blot analysis. Our results further highlight the need for a F4P approach for validation of antibodies before they are placed on the market or discontinued, and we urge suppliers to leave final validation of antibodies for specific end uses to basic researchers.

## Supplementary Information

Below is the link to the electronic supplementary material.

Supplementary file1 (PDF 19476 KB)

## Data Availability

The authors confirm that the data supporting the findings of this study are available within the article and the associated Electronic supplementary material.
