# Supplementary material for: Fit-for-purpose based testing and validation of antibodies to amino- and carboxy-terminal domains of cannabinoid receptor 1
Source: Histochem Cell Biol. 2021 Aug 27;156(5):479–502. doi: 10.1007/s00418-021-02025-5 (PMC8604870; doi:10.1007/s00418-021-02025-5)
Supplement: Supplementary file 1 — Supplementary file1 (PDF 19476 KB) [file 418_2021_2025_MOESM1_ESM.pdf]

Fit-For-Purpose Based Specificity Analysis of Antibodies to Amino- and Carboxy-Terminal Domains of Cannabinoid Receptor 1

Leyre Echeazarra[1,9], Gontzal García del Caño[2,8,*], Sergio Barrondo[3,7,8], Imanol González-Burguera[2,8], Miquel Saumell-Esnaola[3,8], Xabier Aretxabala[2], Maider López de Jesús[3,8], Leire Borrega-Román[3,8], Susana Mato[4,10,11,12], Catherine Ledent[6], Carlos Matute[4,10,11], María Aranzazu Goicolea[5], Joan Sallés[3,7,8,*]

[1] Departament of Physiology, Faculty of Pharmacy, University of the Basque Country UPV/EHU, Vitoria-Gasteiz, Spain.

[2] Department of Neurosciences, Faculty of Pharmacy, University of the Basque Country UPV/EHU, Vitoria-Gasteiz, Spain.

[3] Department of Pharmacology, Faculty of Pharmacy, University of the Basque Country UPV/EHU, Vitoria-Gasteiz, Spain.

[4] Department of Neurosciences, Faculty of Medicine and Nursing, University of the Basque Country UPV/EHU, Leioa, Spain.

[5] Department of Analytical Chemistry, Faculty of Pharmacy, University of the Basque Country UPV/EHU, Vitoria-Gasteiz, Spain.

[6] 6IRIBHN, Universite Libre de Bruxelles, Bruxelles, Belgium.

[7] Centro de Investigación Biomédica en Red de Salud Mental (CIBERSAM), 28029 Madrid, Spain.

[8] Bioaraba, Neurofarmacología Celular y Molecular, 01008 Vitoria-Gasteiz, Spain.

[9] Bioaraba, Dispositivos Móviles para el Control de Enfermedades Crónicas, 01008 Vitoria-Gasteiz, Spain.

[10] Centro de Investigación Biomédica en Red sobre Enfermedades Neurodegenerativas (CIBERNED), Madrid, Spain.

[11] Achucarro Basque Center for Neuroscience, Leioa, Spain.

[12] Biocruces Bizkaia, Multiple Sclerosis and Other Demyelinating Diseases Unit, Barakaldo, Spain.

*** Correspondence:**

Gontzal García del Caño (gontzal.garcia@ehu.eus)

Joan Sallés (joan.salles@ehu.eus)

Supplementary Materials and Methods

Isolation of enriched subcellular fractions

To obtain P1, P2 and cytosolic (Cyt) subcellular fractions, brain tissue samples were thawed, homogenized in 20 volumes of ice-cold Tris/EGTA buffer (20 mM Tris-HCl buffer, pH 7.4, 1 mM ethylene glycol-bis(β-aminoethyl ether)-N,N,N′,N′-tetraacetic acid, 1 mM phenylmethylsulfonyl fluoride and 0.5 mM iodoacetamide) and centrifuged at 1,100 × *g* for 10 min. The pellet washed once more at 1,100 × *g* for 10 min in 320 mM sucrose-containing fresh Tris/EGTA buffer to obtain the P1 fraction and the supernatant was centrifuged at 40,000 × *g* for 10 min to obtain a supernatant (Cyt fraction) and a pellet, which was washed twice more with fresh Tris/EGTA buffer at 40,000 × *g* for 10 min to obtain the final pellet corresponding to P2 fraction.

To isolate highly purified intact nuclei (N fraction), cerebral cortices were dissected from five adult rats and six adult mice (3 CB_1-WT and 3 CB_1-KO). A small piece of the three cerebral cortices of each mouse phenotype was set aside and frozen for subsequent reverse transcriptase polymerase chain reaction (RT-PCR) testing (see below). Tissue samples were chopped finely in 1 mM $MgCl_2$, containing 2.0 M sucrose and protease inhibitors (1 mM phenylmethylsulfonyl fluoride and 0.5 mM iodoacetamide) and homogenized to give a 20% (w/v) homogenate. The homogenate was then filtered through one layer of muslin and centrifuged at 4 °C for 60 min at 64,000 × *g* in a SW40Ti rotor (331302; Beckman). The obtained pellet was resuspended in 1 mM $MgCl_2$, containing 320 mM sucrose and protease inhibitors, and centrifuged for 5 min at 1,500 × *g* to obtain N fraction). Nuclei used for immunofluorescence analysis were resuspended in 10 mM Tris–HCl, pH 7.2 containing 2 mM $MgCl_2$ at a dilution of 2×10^6 nuclei/ml. Then, 25 µl drops were laid on gelatine-coated slides, allowed to dry at room temperature, and stored at -80 °C until use. Total protein concentration was estimated in all samples with the Bradford protein assay kit #5000006 (Bio-Rad Laboratories, Madrid, Spain) using bovine γ-globulin as standard.

Western blotting

Blots were blocked in 5% non-fat dry milk/PBS containing 0.5% BSA and 0.2% Tween-20 for 1 h, and incubated (overnight at 4 °C in blocking buffer without milk) with primary antibodies against CB1 receptor, against G inhibitory protein alpha subunits 1-3 (Gαi-1,2,3), or against several proteins specific to particular subcellular compartments: [i] 62-kDa component (Nup62) of the nuclear pore complex (NPCx) and histone H1 as a nuclear

markers, [ii] α1 subunit of Na+/K+ ATPase, NR1 subunit of the NMDA receptor (NMDAR1) and synaptosome-associated protein 25 (SNAP25) as markers of the plasma membrane fraction and [iii] β-tubulin as a cytosolic marker (Supplementary Table 1 for details). Blots were washed and incubated with specific horseradish peroxidase conjugated secondary antibodies diluted to 1:10000 in blocking buffer for 2 h at 20–25 °C. Blots were washed and incubated with horseradish peroxidase (HRP) conjugated secondary antibodies HRP-conjugated Rabbit anti-Goat IgG (A5420; Sigma-Aldrich), HRP-conjugated donkey anti-Rabbit IgG (NA934; Amersham Biosciences) or HRP-conjugated Sheep anti-Mouse IgG (NXA931; Amersham Biosciences), all diluted to 1:10,000 in blocking buffer for 2 h at 20–25 °C. Immunoreactive bands were visualized with Clarity Western ECL Substrate (#1705061; Bio-Rad Laboratories) according to the manufacturer instructions. A colour prestained broad-range protein ladder (MB090, NZYtech, Lisbon, Portugal) to estimate the molecular mass of individual bands.

Binding assays

Saturation binding experiments with the selective CB_1 receptor antagonist [^{3}H]SR141716A were carried out in P1, P2 and N subcellular fractions of the adult rat brain cortex. Non-specific binding was determined in the presence of 10 μM WIN 55,212-2. Assays were performed by incubating suspensions of P1, P2 and N samples (0.2 mg protein /ml) with increasing concentrations of [^{3}H]SR141716A (triplicates of 10 different concentrations, from 0.01 to 10 nM) at 37 °C in 50 mM Tris-HCl (pH 7.4) during 1 hour. Following the incubation with the radioligand, membranes were harvested by washing twice with 4 ml of ice-cold binding buffer (4 °C). Membrane bound radioligand was measured by rapid filtration under vacuum through Whatman GF/C glass fibre filters, which had been presoaked with binding buffer during two hours. Individual filters were transferred to scintillation vials containing 4 ml of Optiphase HiSafe® and counted for radioactivity by liquid scintillation spectrometry (Packard model 2200 CA).

For assays of [^{35}S]GTPγS binding stimulated by WIN 55, 212-2, P1 and P2 membranes from the adult brain cortex were thawed and incubated at 30 °C for 2 h in [^{35}S]GTPγS-incubation buffer (0.5 mM [^{35}S]GTPγS, 1 mM EGTA, 3 mM $MgCl_2$, 100 mM NaCl, 0.2 mM DTT, 50 μM GDP, and 50 mM Tris-HCl, pH 7.4). Increasing concentrations of the CB_1 cannabinoid receptor agonist WIN 55, 212-2 (10^{-10}–10^{-4} M) were added to determine receptor-stimulated [^{35}S]GTPγS binding. Non-specific binding was defined in the presence

of 10 µM unlabelled GTPγS. Basal binding was assumed to be the specific [^{35}S]GTPγS binding in the absence of agonist. The reactions were terminated by rapid vacuum and filtration through Whatman GF/C glass fibre filters and the remaining bound radioactivity was measured by liquid scintillation spectrophotometry.

Reverse-Transcriptase Polymerase Chain Reaction and Sequencing

Frozen cerebral cortex samples from Ledent's line; CB_1-WT and CB_1-KO mice were lysed in Trizol (15596-026; Invitrogen S.A.), followed by total RNA purification by aqueous phase separation in chloroform and subsequent precipitation with isopropyl alcohol and centrifugation at 14,000 rpm. The RNA-containing pellet was washed with 75% ethanol 0.1% diethylpyrocarbonate treated ultrapure H_2O (DEPC water), recovered from a 8,500 rpm pellet, allowed to dry, dissolved in DEPC water and treated with Ambion™ DNase I (RNase-free) (AM2222; Invitrogen S.A.) to remove any DNA contamination. The yield and quality of the RNA were assessed by measuring absorbance at 260, 270, 280 and 310 nm and by electrophoresis on 1.3% agarose gels. From each sample, 1.5 µg total RNA was reverse-transcribed to first-strand complementary DNA (cDNA) with iScript™ cDNA Synthesis Kit (#1708891; Bio-Rad). The cDNA was subjected to PCR amplification by three primer pairs (Integrated DNA Technologies S.L., Madrid, Spain) targeting different regions of the mouse Cnr1 gene: CB1-A (Fw, CGTTGAGCCTGGCCTAATCA; Rv, AACCAACGGGGAGTTGTCTC), CB1-B (Fw, TGTGGGCAGCCTGTTCCTCA; Rv, CATGCGGGCTTGGTCAGG) and CB1-C (Fw, GATGTCTTTGGGAAGATGAACAAGC; Rv, GACGTGTCTGTGGACACAGACATGG). Briefly, 2 µL of 10 and 100-fold diluted cDNA were amplified in a final volume of 50 µL reaction mixture containing 2 mM Mg^{2+}, 0.3 mM dNTPs, 0.3 µM Fw and Rv primers and 1.5 units KAPA HiFi Taq polymerase (KK2101; Kapa Biosystems, Inc., Woburn, MA, USA). After optimising the annealing conditions using a temperature gradient (61.0, 63.8, 66.2, 69.6 °C), all PCR reactions were run at 94 °C for 5 min, followed by 30 amplification cycles consisting of melting at 96 °C for 1 min, annealing at 66 °C for 30 sec and extension at 72 °C for 1 min, followed by a final extension step at 72 °C for 5 min. The PCR products were run in agarose gels, followed by purification of the amplicons of interest using Macherey-Nagel Nucleospin® Gel and PCR Clean-Up (740609.250; Clontech, Madrid, Spain) according to the manufacturer's instructions. The purified PCR products were inserted into the pCR™-Blunt II-TOPO™ cloning plasmid, using the Zero Blunt™ TOPO™ cloning kit (K280002; ThermoFisher Scientific, Barcelona, Spain) following the manufacturer's instructions. After heat shock transformation One Shot®

TOP10 chemically competent bacteria (C404010; Invitrogen) with the PCR product-containing plasmid five clones containing insert (as determined by restriction enzyme analysis) were chosen for sequencing at the STABVIDA Sequencing Facilities (Lisbon. Portugal).

Supplementary results

Effect of sulphide fixation on the immunostaining pattern produced by Af380 and Af450 antibodies in adult rat cortex

When tissue sections of the adult rat cortex immunostained with the anti-CB_1 Af380 antibody Af380 under sulphide and standard fixation conditions were compared, no obvious qualitative differences were observed in the gross distribution of immunoreactivity. Thus, in either condition, Af380 antibody labelled axonal profiles and presynaptic-like puncta, which were distributed in a layer-specific fashion throughout the depth of neocortex. As previously described (Egertová_&_Elphick, 2000; Bodor et al, 2005, Deshmukh et al., 2007), axonal staining density was highest in layers II/III, followed by layers VI, IV and V (Fig. S3). Despite the similar overall distribution of immunostaining between the two conditions, fibre profiles and presyntaptic-like puncta were more intensely stained under sulphide fixation and some subtle but evident differences were observed in the density of immunostained fibre profiles. Particularly obvious, a dense plexus of fibre profiles and presynaptic-like varicosities could be observed in the upper third of layer II/III in either of the two conditions (Figs. S3a, e); however, the fibre density decreased abruptly in the lower two-thirds of layers II / III under standard conditions, but not under sulphide fixation conditions but not under sodium sulphide fixation (Fig. S3e). In layer V of sections fixed by the standard method, axonal profiles were hardly distinguishable from the background staining, whereas neuronal somata stood out clearly against the surrounding tissue (Fig. S3c). By contrast, under sulphide fixation, a loose but clearly distinguishable network of axons emerged in layer V, whereas a pale staining hardly distinguishable against the background was observed in some perikarya (Fig. S3f). Similar to rabbit Af350 antibody, goat Af450 antibody produced a clear axonal and presynaptic-like immunostaining pattern with a similar layer-specific distribution under both standard and sulphide fixation conditions (Fig. S4a). However, while under standard conditions immunostaining was largely restricted to fibre profiles and presynaptic-like puncta (Figs. S4a-c), a conspicuous somatic immunostaining composed of round profiles that resembled cell nuclei was clearly observed in cortical tissue sections from brains subjected

to sulphide fixation (Figs. S4d-f), this pattern difference being particularly obvious in layer V but also observable in layers II/III (Compare Figs. S4c-d and S4e-f).

Reverse-Transcriptase Polymerase Chain Reaction and Sequencing

Brain cortex samples from Ledent's CB_1-WT and CB_1-KO mice were processed for total RNA purification, checked for quality by spectrometry (Fig. S8a) and agarose gel electrophoresis (Fig. S8b) and used to generate cDNA by reverse transcription. Then, PCR amplification was carried out with three different primers pairs to rule out the possibility that a transcript containing the coding sequence for the immunizing peptide (residues 443-473 of mouse CB_1 receptor) could be still expressed in CB_1-KO mice: [i] a first one (CB1-A) with both primers annealing only within the Cnr1 gene sequence of the CB_1-WT mice, [ii] a second one (CB1-B) consisting of a forward primer complementary to a sequence of the Cnr1 gene only present in the CB_1-WT mice and a reverse primer complementary to a position present in both phenotypes, and [iii] a third one (CB1-C) with both primers complementary to sequences of the Cnr1 gene common to both phenotypes (Fig. S9b). As expected, CB1-A and CB1-B primer pairs yielded PCR products of the expected size only in cDNA samples from CB_1-WT mice, whereas CB1-C pair gave rise to several products of different size in both CB_1-WT and CB_1-KO phenotypes, some of which were consistent with the expected theoretical size of 308 bp. However, sequencing showed that only the PCR product detected in the CB_1-WT mice corresponded to mouse Cnr1 gene sequence (NCBI Accession: NM_007726.5) sharing 100% nucleotide identity, whereas the PCR product detected in CB_1-KO mice consisted of a 296 nt sequence with 100% homology with two non-contiguous sequences of the mouse Actr6 gene transcript (Ensembl: MGP_C57BL6NJ_T0027247.1), coding for Actin-related protein 6 (UniProtKB: Q9D864), consisting of a 255 nt sequence at the 3'-end of intron 4-5 followed by a 41 nt fragment within exon 11 of the transcript. Analysis of 5' and 3' ends of the non-specific PCR amplicon indicated that it was derived from pairing of truncated primers rather than from illegitimate pairing of full-length PCR primers. Indeed, Fw and Rv primers of the CB1-C pair were 5 to 7 nt longer than the rest and, since the coupling efficiency of DNA synthesis is <100% per base, more truncated oligonucleotides accumulate during synthesis.

Primary antibodies used for dobule IF and WB						
**Recognizing protein**	**Dilution (IF)**	**Dilution (WB)**	**Host and clonality**	**Isotype and purity**	**Immunizing antigen and recognised epitope (when available)**	**Source, Catalog No.**
Lamin B1	1:100		Mouse monoclonal (clone 8D1)	Affinity-purified kappa light chain of IgG_1	Purified cell nuclei from HeLa human epithelioid carcinoma.	Sta Cruz Biotech., 8D1: sc-56144
$G\alpha_{i-1/2/3}$		1:100	Goat polyclonal	Affinity purified serum	Peptide mapping near the N-terminus of $G\alpha_{i-1}$ of human origin, common to $G\alpha_{i-1}$, $G\alpha_{i-2}$ and $G\alpha_{i-3}$.	Sta Cruz Biotech., N-20: sc-26761
H1 Histone	1:500	1:200	Mouse monoclonal (clone AE-4)	Affinity-purified kappa light chain of IgG_{2a}	Leukemia biopsy cells of human origin.	Sta Cruz Biotech., AE-4: sc-8030
Na^+/K^+ ATPase		1:5000	Mouse monoclonal (clone M8-P1-A3)	Immunogen affinity-purified IgG_1	Peptide corresponding to amino acids 496–506 of Na^+/K^+ ATPase α_1 subunit from lamb kidney.	Sigma-Aldrich, A277
NeuN/Fox-3	1:1000		Mouse monoclonal (clone A60)	Affinity-purified IgG_1	Purified cell nuclei from mouse brain	Millipore, MAB377
NMDAR1		1:1500	Rabbit polyclonal	Affinity purified serum	Non-phosphopeptide peptide around the phosphorylation site at serine 897 of the human NR1 subunit of the NMDA receptor.	Abcam, ab52177
NPCx		1:5000	Mouse monoclonal (clone Mab414)	Immunogen affinity-purified IgG_1	Nuclear pore complex mixture. Recognizes the conserved domain FXFG repeats in nucleaporins like the p62, p152, p90.	Abcam, ab24609
SC35	1:500		Mouse monoclonal (clone SC-35)l	Immunogen affinity-purified IgG_1	Phospho-peptide corresponding to an epitope of the non-snRNP factor SC35.	Abcam, ab11826
SNAP25		1:4000	Mouse monoclonal (clone SP12)	IgG_1 from mouse ascites	Crude synaptic preparation from the postmortem human brain.	Abcam, ab24732
β-tubulin		1:500	Mouse monoclonal (clone TUB2.1)	IgG_1 from mouse ascites	Purified rat brain tubulin. Recognizes an epitope in the carboxy-terminal part of all five β-tubulin isoforms (β1-β5).	Sigma-Aldrich, T4026

Suplementary Table 1 IF, immunofluorescence; **WB**, Western blot**. Antibody manufacturers:** Abcam, Cambridge, UK; Millipore, Billerica, MA, USA; Santa Cruz Biotechnology, Santa Cruz, CA, USA; Sigma-Aldrich, St. Louis, MO, USA.

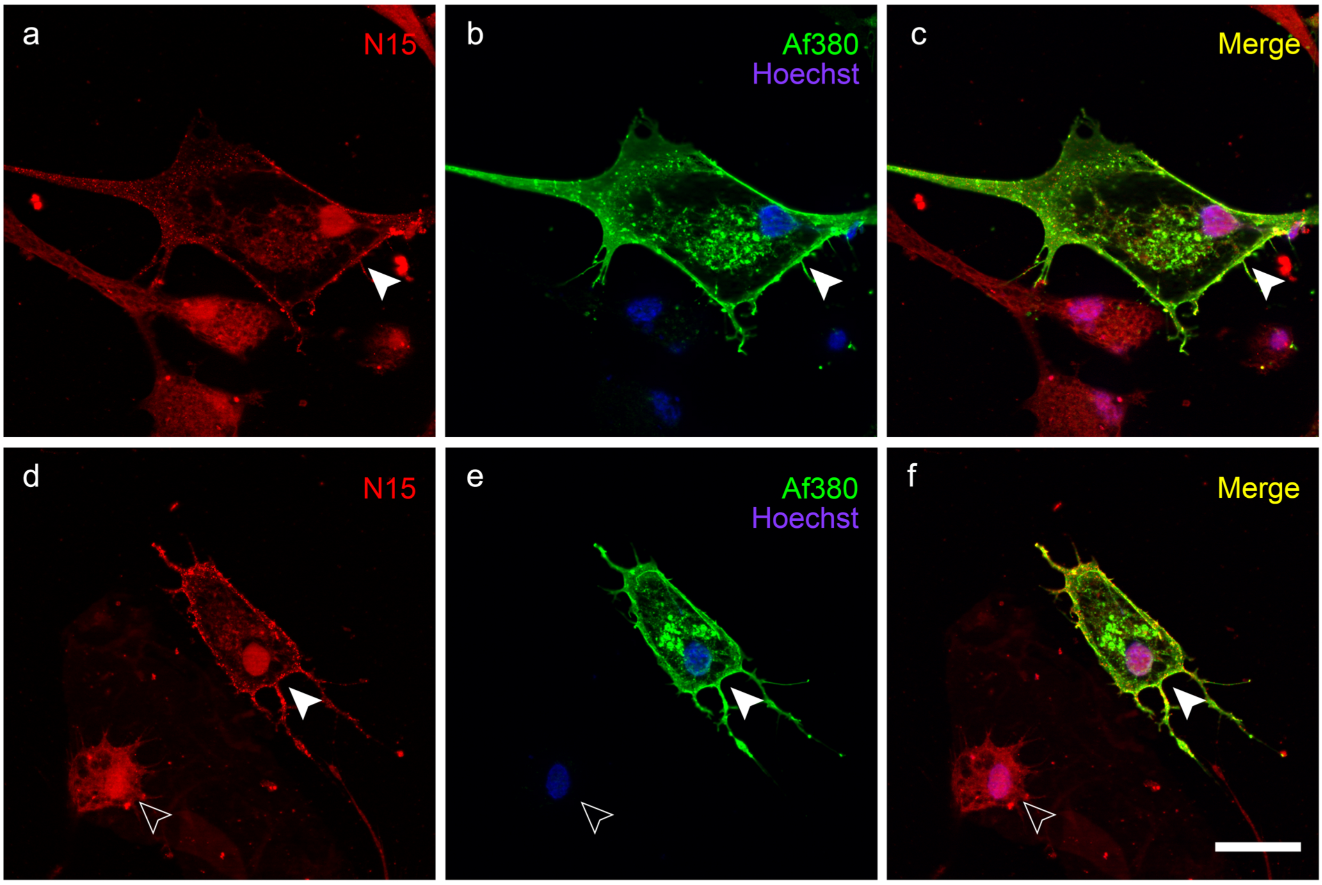

Supplementary Fig. S1 Live immunolabelling of in CB_1-transfected HEK293 cells with the N-terminal N15 (pseudocoloured red) and the C-terminal Af380 antibodies (pseudocoloured green) showing the ability of N15 antibody to detect plasma membrane staining in some cells, but with a high background autofluorescence due to the long exposure time needed to observe surgace staining. Single-channel images shown in a-b and d-e were merged together with Hoechst's chromatin staining (shown in c and f, respectively). Micrographs are maximum intensity projections of 3 consecutive optical sections separated by 0.24 μm, obtained by structured illumination microscopy. Scale bar: 20 μm (applies to a-f).

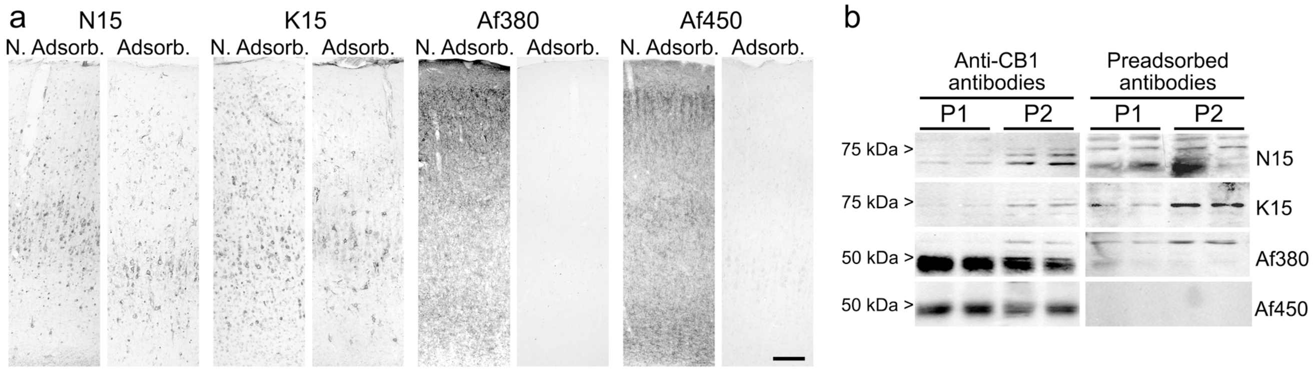

Supplementary Fig. S2 a. Low magnification Micrographs showing immunohistochemical staining pattern using anti-CB_1 antibodies before and after being preadsorbed with the corresponding blocking peptide. Scale bar: 150 µm (applies to all captures shown in a). **b**. Western blot analysis using anti-CB_1 antibodies before and after preadsorption with the corresponding antigenic sequence. Equivalent amounts of protein (20 µg/lane) from P1 and P2 fractions obtained from homogenates of adult rat brain cortex were loaded in duplicate and run in parallel. The H150 rabbit polyclonal antibody was not included in these assays because a blocking peptide was not available.

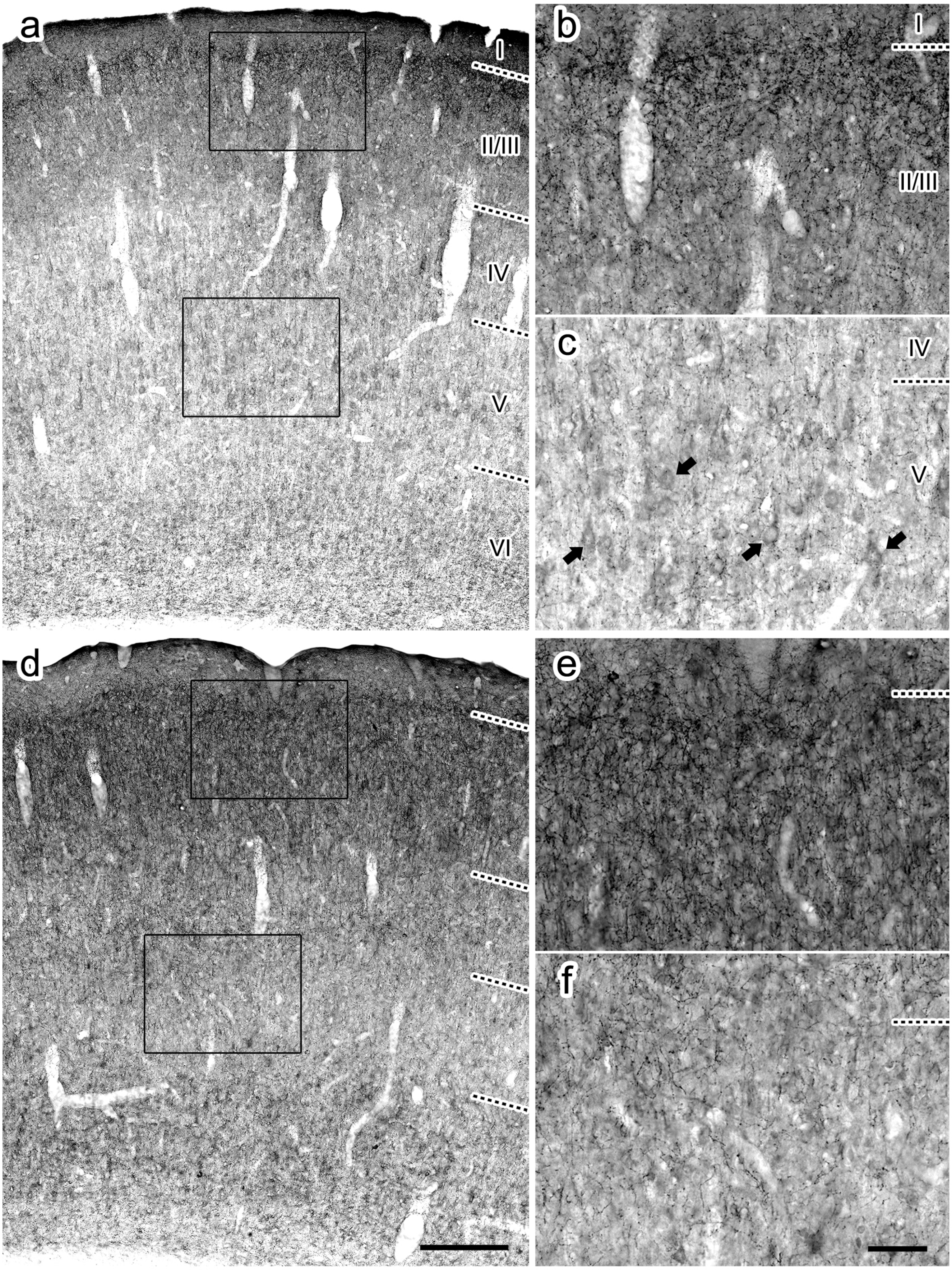

Supplementary Fig. S3 Immunohistochemical staining with the rabbit polyclonal Af380 anti-CB_1 antibody in the adult rat parietal cortex under either standard (**a-c**) or sodium sulphide (**d-f**) fixation. Panoramic micrographs showing the distribution of immunoreactivity throughout cortical layers (a, d) and higher magnification micrographs of framed areas in a and d (b-c and e-f, respectively). Broken lines indicate the boundaries of cortical layers I-VI. Arrows show immunostained perikarya in layer V. Scale bars: 200 µm in d (applies to a, d); 50 µm in f (applies to b-c, e-f).

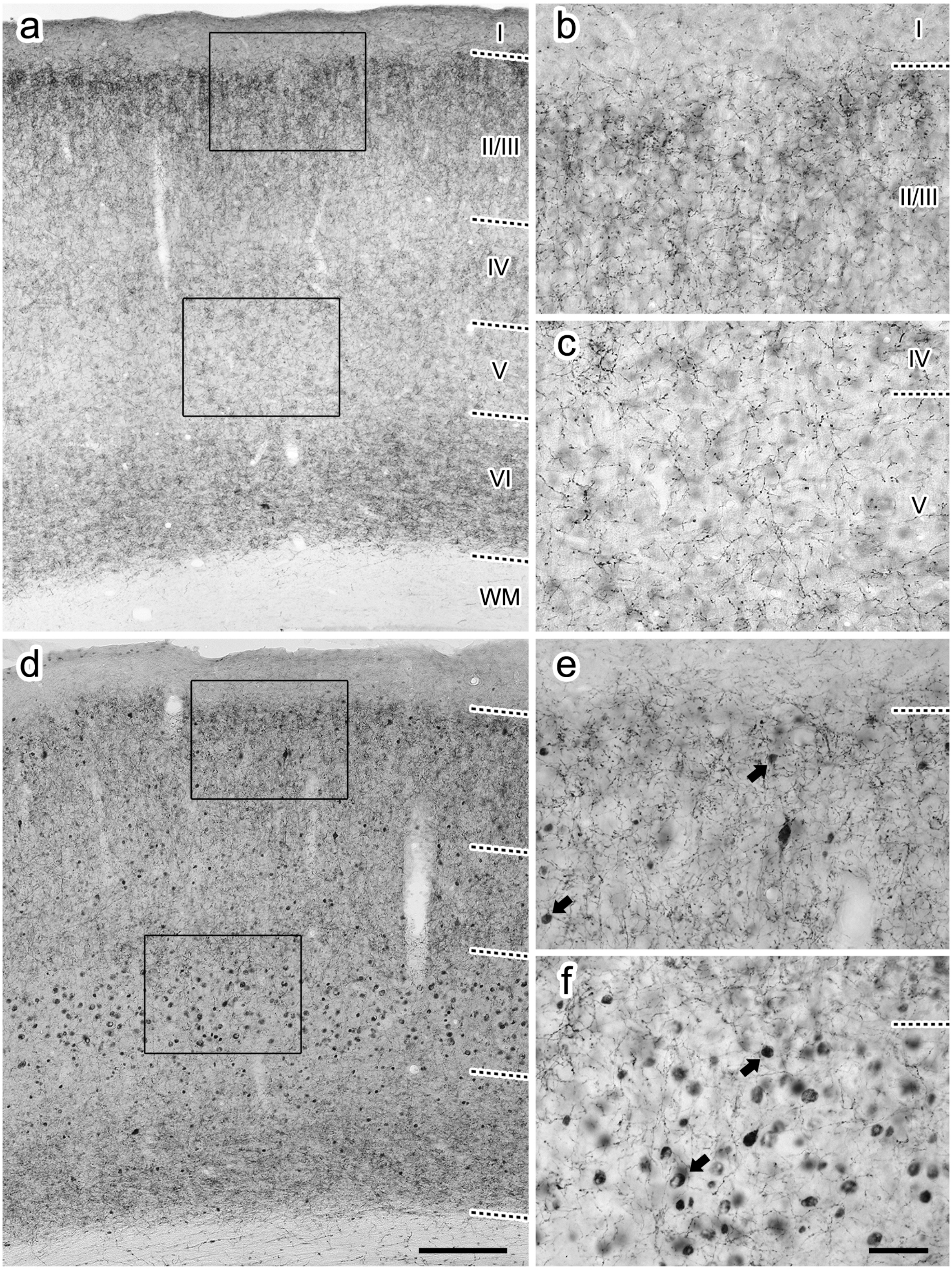

Supplementary Fig. S4 Immunohistochemical staining with the goat polyclonal Af450 anti-CB_1 antibody in the adult rat parietal cortex under either standard (**a-c**) or sodium sulphide (**d-f**) fixation. Panoramic micrographs showing the distribution of immunoreactivity throughout cortical layers (a, d) and higher magnification micrographs of framed areas in a and d (b-c and e-f, respectively). Broken lines indicate the boundaries of cortical layers I-VI. Arrows show somatic staining observed clearly under sulphide fixation. Scale bars: 200 μm in d (applies to a, d); 50 μm in f (applies to b-c, e-f).

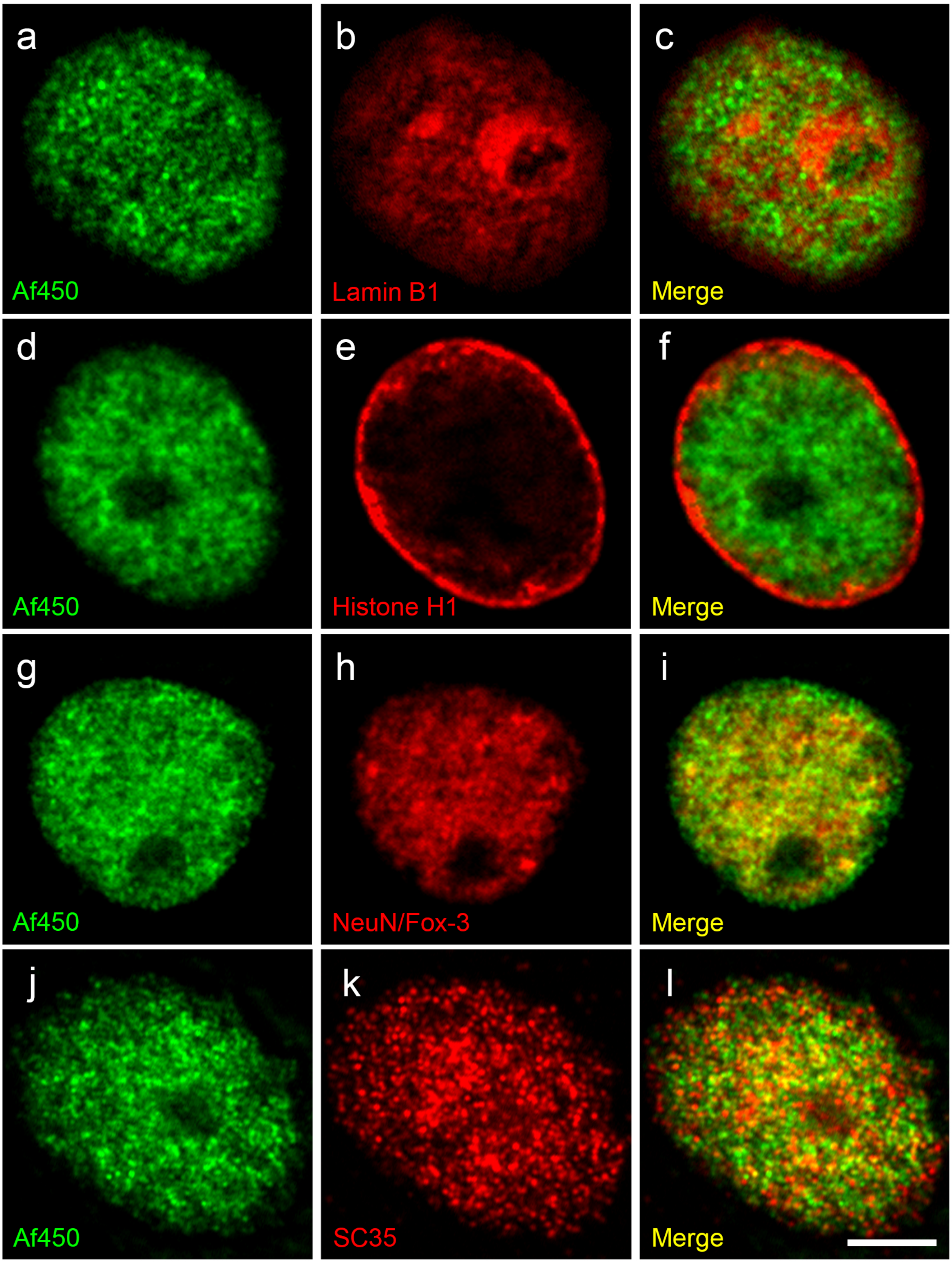

Supplementary Fig. S5 High-resolution fluorescence microscopy images showing the distribution of immunoreactivity detected in neuronal nuclei isolated from the adult rat brain using the anti-CB_1 Af450 antibody. Nuclei laid on poly-L-ornithine-coated coverslips and subjected to double immunofluorescence by combining Af450 with antibodies against histone H1 (**a-c**), lamin B1 (**d-f**), NeuN/Fox-3 (**g-i**) or SC35 (**j-l**). Af450 immunoreactivity was distributed throughout histone H1-negative subdomains of the nucleoplasm (a-c), internal to the nuclear lamina (d-f) and colocalizing partially with the nuclear matrix and nuclear speckle markers NeuN/Fox-3 (g-i) and SC35 (j-l). Micrographs are single 0.5 μm-thick optical sections obtained by structured illumination microscopy. Scale bar: 5 μm in l (applies to a-l).

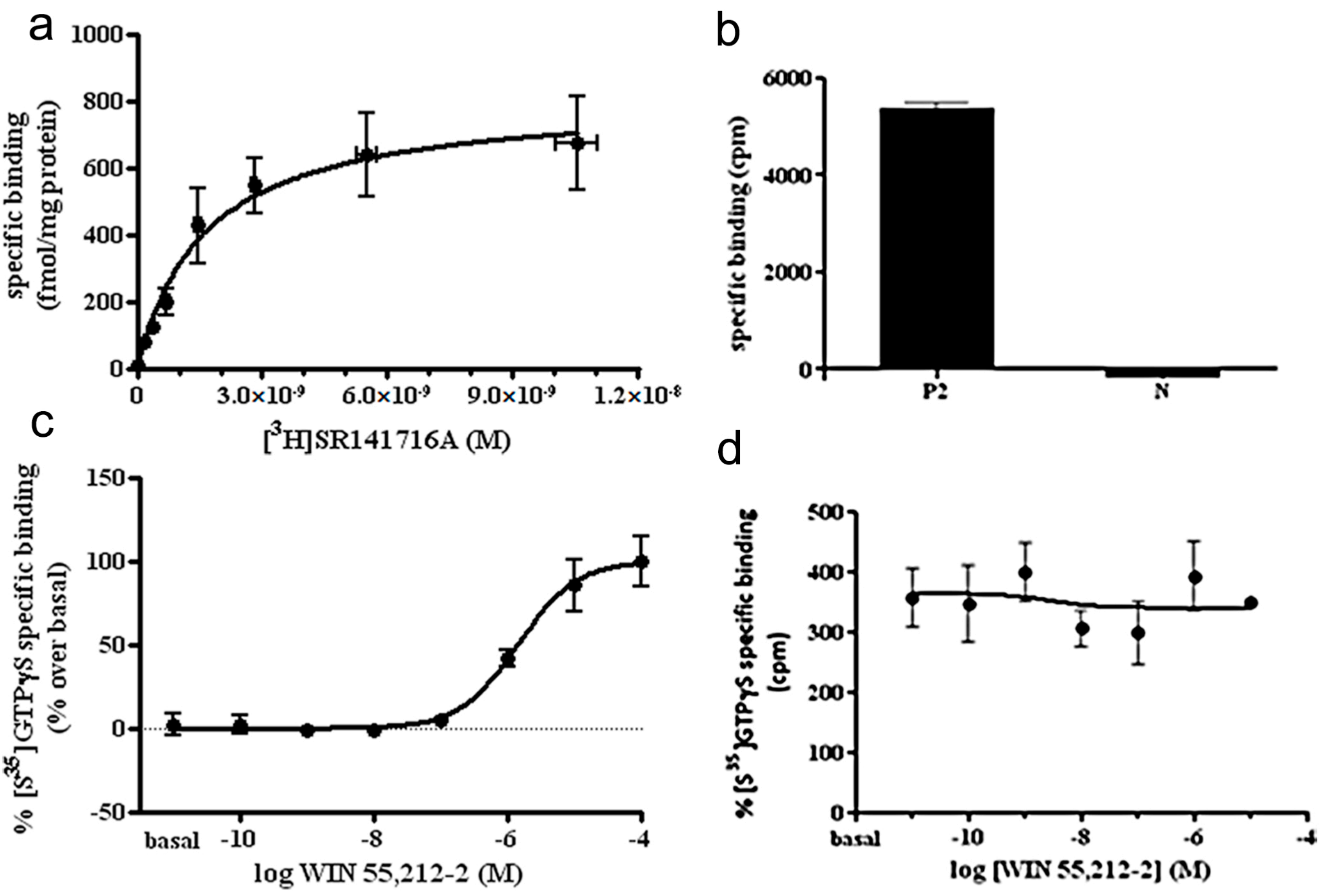

Supplementary Fig. S6 [^{3}H]SR141716A radioligand binding (**a-b**) and CB_1 receptor agonist-stimulated [^{35}S]GTPγS binding (**c-d**) assays in P2 and N fractions from the adult rat brain cortex. Assays were performed in conditions identical to those described in figure 6. Each point in saturation and [^{35}S]GTPγS stimulation curves correspond to the mean ± SEM values of a representative experiment performed in triplicate and duplicate, respectively. Note that neither [^{3}H]SR141716A binding nor WIN 55,212-2 agonist-stimulated [^{35}S]GTPγS binding could be observed in samples of intact nuclei.

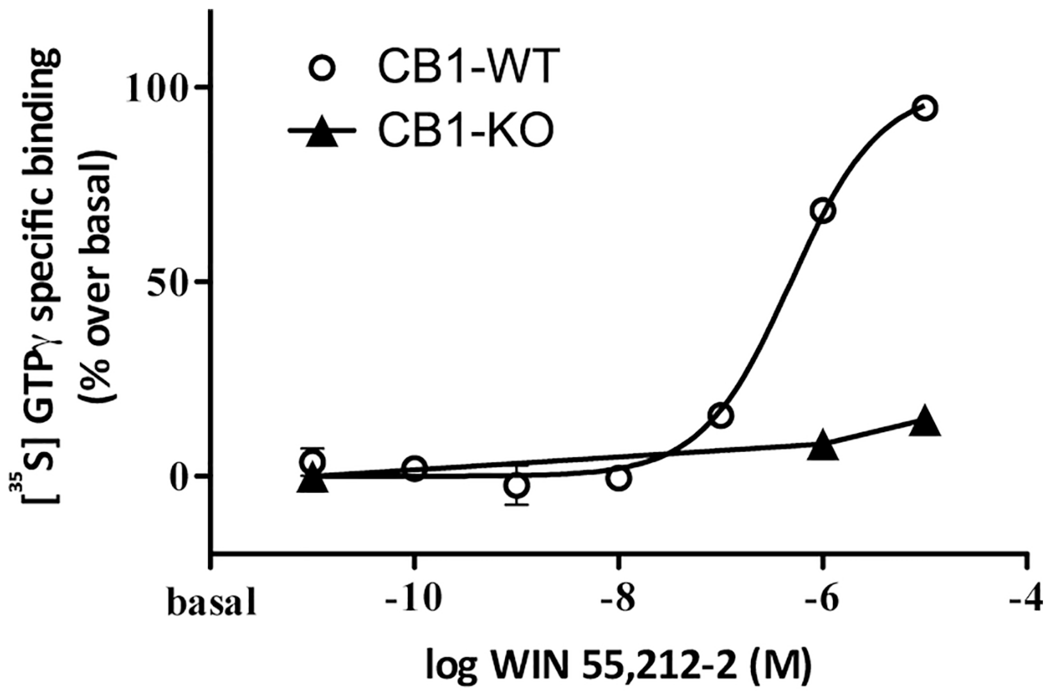

Supplementary Fig. S7 CB_1 receptor agonist-stimulated [^{35}S]GTPγS binding assays in P2 samples from adult mouse brain cortex of CB_1-WT and CB_1-KO mice of Ledent's line. [^{35}S]GTP γS binding assays were performed in conditions identical to those described in figure 6. Each point in the stimulation curves correspond to the mean ± SEM values of a representative experiment performed in duplicate. WIN 55,212-2 agonist-stimulated [^{35}S]GTPγS binding could be only observed in P2 samples isolated from CB_1-WT animals.

a

	CB1-WT	CB1-KO
**$Abs_{260/280}$**	2.03	2.06
**$Abs_{260/270}$**	1.24	1.24
**Abs_{310}**	0.006	-0.012
**[RNA] = µg/µl**	13,6	14,4

b

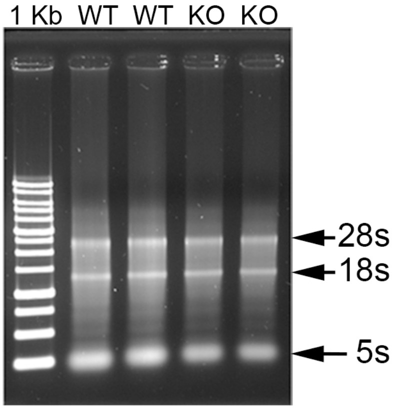

Supplementary Fig. S8 Spectrophotometry and agarose gel electrophoresis analysis of RNA isolated from the adult brain cortex of CB_1-WT and CB_1-KO mice. **a**. $Abs_{260/280}$ and $Abs_{260/270}$ ratio values of ~2 and 1.2, respectively, along with Abs_{310} value very close to zero, were indicative of the purity of RNA and of the absence of protein and/or phenol contamination. RNA concentration was calculated from the absorbance at 260 nm **b**. RNA was resolved by agarose gel electrophoresis, stained with GelRed® (41002; Biotium Inc., Biotium, Hayward, USA) and visualized under UV light. Note the integrity of the 5S, 18S and 28S ribosomal RNA bands and the absence of bands above the signal corresponding to the 28s subunit.

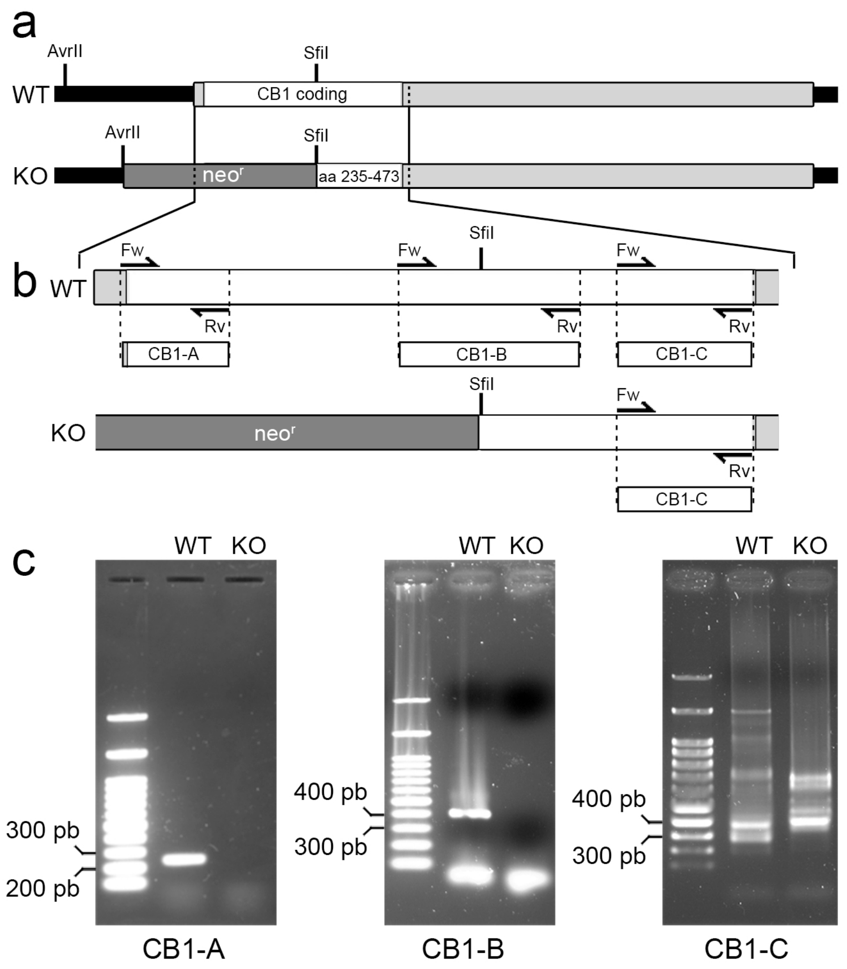

Supplementary Fig. S9 PCR analysis of the presence of Cnr1 transcript in brain cortex samples from Ledent's CB_1-WT and CB_1-KO mice. **a**. Schematic representation of the murine CB_1 receptor gene (Cnr1) and the allelic variant of the CB_1-KO mouse generated by homologous recombination by Ledent and colleagues (Ledent et al., 1999). Mouse Cnr1 gene is composed of a short 5' noncoding region (light grey), followed by a single known coding exon (white box) and a long 3' noncoding sequence (light grey). In CB_1-KO mice, a PGK-neo^r cassette replaces a DNA fragment encompassing a sequence between AvrII and SfiI restriction sites. The removed sequence includes the 5' non-coding sequence of Cnr1 gene and the 5' end of the coding sequence, thus leading to a null allele that still contains the triplets of Cnr1 gene coding for amino acids 234-472. **b**. Schematic representation of the Cnr1 transcript in CB_1-WT mice and of a potential transcript derived from the null allele of the CB_1-KO mouse containing the 3' end of the Cnr1 coding sequence. The potential binding sites of the primer pairs used and the resulting transcripts are schematically shown. CB1-A Fw/CB1-A Rv pair was designed using Primer3 software (Rozen and Skaletsky, 2000). CB1-B Fw/CB1-B Rv pair was designed based on the sequence of HCB1U and HCB1L primers, widely used to amplify human CNR1 transcript (Jiang et al., 2007; Moaddel et al, 2011) with a single base modification to achieve full homology with the mouse sequence. The sequence of CB1-C Fw/CB1-C Rv pair was obtained from Sarnataro et al. (2006). **c**. Results of PCR amplification using cDNA obtained by reverse transcription from brain cortex of CB_1-WT and CB_1-KO mice as matrices. Signal was obtained after agarose gel-electrophoresis of PCR products (1 µl/lane) GelRed® staining.

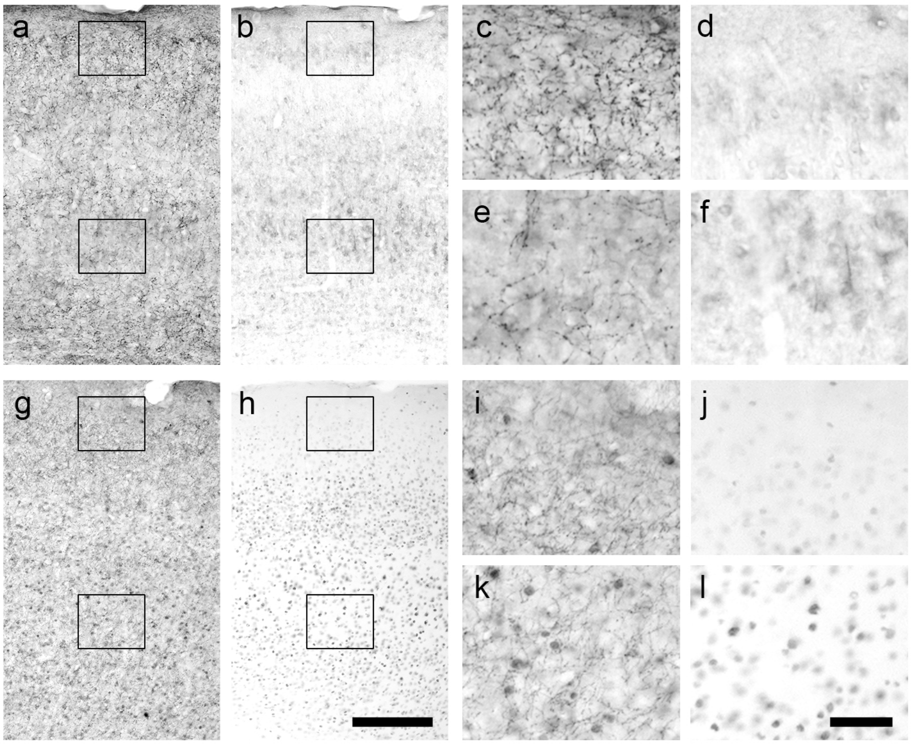

Supplementary Fig. S10 Anti-CB_1 receptor immunohistochemical staining in sulphide-fixed parietal cortex sections from CB_1-WT and CB_1-KO mice of the Marsicano's line using the rabbit polyclonal Af380 (**a-f**) and the goat polyclonal Af450 (**g-l**) antibodies in the parietal cortex of CB_1-WT (a, c, e) and CB_1-KO (b, d, f) mice. Framed areas in panoramic images a, b, g and h are shown at higher magnification in c-d, e-f, i-j and k-l, respectively. Scale bars: 200 µm in h (applies to a-b, g-h), 50 µm in l (applies to c-f, i-l).
